# Biomimetic cell membrane‐coated poly(lactic‐ *co* ‐glycolic acid) nanoparticles for biomedical applications

**DOI:** 10.1002/btm2.10441

**Published:** 2022-11-02

**Authors:** Nasrullah Jan, Asadullah Madni, Safiullah Khan, Hassan Shah, Faizan Akram, Arshad Khan, Derya Ertas, Mohammad F. Bostanudin, Christopher H. Contag, Nureddin Ashammakhi, Yavuz Nuri Ertas

**Affiliations:** ^1^ Akson College of Pharmacy Mirpur University of Science and Technology (MUST) Mirpur Pakistan; ^2^ Department of Pharmaceutics, Faculty of Pharmacy The Islamia University of Bahawalpur Bahawalpur Pakistan; ^3^ Department of Biomedical Engineering Erciyes University Kayseri Turkey; ^4^ College of Pharmacy Al Ain University Abu Dhabi United Arab Emirates; ^5^ AAU Health and Biomedical Research Center Al Ain University Abu Dhabi United Arab Emirates; ^6^ Department of Microbiology and Molecular Genetics Michigan State University East Lansing Michigan USA; ^7^ Institute for Quantitative Health Science and Engineering (IQ) and Department of Biomedical Engineering (BME) Michigan State University East Lansing Michigan USA; ^8^ Department of Bioengineering University of California, Los Angeles Los Angeles California USA; ^9^ ERNAM–Nanotechnology Research and Application Center Erciyes University Kayseri Turkey; ^10^ UNAM–National Nanotechnology Research Center Bilkent University Ankara Turkey

**Keywords:** biodegradable, biomimetic, cell‐membrane, drug delivery, PLGA, targeted delivery

## Abstract

Poly(lactic‐*co*‐glycolic acid) (PLGA) nanoparticles (NPs) are commonly used for drug delivery because of their favored biocompatibility and suitability for sustained and controlled drug release. To prolong NP circulation time, enable target‐specific drug delivery and overcome physiological barriers, NPs camouflaged in cell membranes have been developed and evaluated to improve drug delivery. Here, we discuss recent advances in cell membrane‐coated PLGA NPs, their preparation methods, and their application to cancer therapy, management of inflammation, treatment of cardiovascular disease and control of infection. We address the current challenges and highlight future research directions needed for effective use of cell membrane‐camouflaged NPs.

## 1 INTRODUCTION

Conventional therapies used for the management of various disease conditions rely on the use of systemic drug administration, which is largely nontargeted to the site of disease.^1^ Consequently, these agents affect both diseased cells in target tissues, and healthy cells at other anatomic sites, leading to unwanted side effects.^2^ Clear examples of the off target effects are seen in cancer chemotherapy, where patients suffer from gastrointestinal (GI) symptoms, anemia, and loss of hair due to the effect of chemotherapy on normal cells, adding problems to patient's life.^3^, ^4^ Because of these serious side effects, the chemotherapeutic dose is kept low to reduce these complications; however, this may necessitate a prolonged course of therapy and even infective treatment with possible disease recurrence^5^, ^6^ and subsequent mortality. Targeted drug delivery has been proposed to address these limitations with the intent of directing drugs to specific tissue sites^7^; this concept holds great promise for increased efficacy using lower doses with subsequent improved therapeutic outcomes and fewer side effects.^2^, ^8^

Recent advances in nanotechnology led to the development of nanoparticles (NPs) that can be decorated with targeting ligands, enzymes, polymers, and other biomolecules for directed delivery. Nanoparticulate delivery systems comprise a paradigm shift, and engineered NPs can have specific physicochemical characteristics by altering surface charge, size, morphology, and surface hydrophilicity for enhanced accumulation in target tissues.^9^, ^10^ Despite significant advances in synthetic NP‐based drug delivery systems, synthetic materials are foreign to the body, and can elicit nonspecific immune reactions.^11^ Other challenges associated with these materials are their premature degradation and release of cargo, leading to rapid renal and hepatic clearance with insufficient drug being available for treatment, effective drug carriers must provide protection to their cargo by preventing premature degradation, prolonging in vivo retention, and protecting from immune surveillance, while preserving controlled drug release at the target site.^12^ The use of biomimetic systems for drug delivery is an approach that may address some of these demands by imbuing cellular functions on synthetic particles.^13^ By recapitulating the shape, movement and surface composition of cells, NPs may evade immune clearance to increase circulation time and protect the drug cargo, and if well‐designed could also preserve the structure to improve drug release at the target site. Such agents would be more effective in vivo drug delivery tools.^14^ Biomimetic systems employ cell membrane‐camouflaged NPs that can be fabricated using top‐down method, where core materials are coated with a cell membrane derived from natural cells.^15^ Using this method, the resulting camouflaged NPs attain special functions including immune escape,^16^ prolonged blood circulation, ligand recognition, and targeting,^17^ which enable a wide range of applications of this system in drug delivery,^15^ photothermal therapy,^18^ vaccination,^19^ and detoxifications.^9^ Core materials used in the systems comprise a variety of materials that include polymeric NPs, silica NPs, gold nanocages, magnetic NPs, and liposomes.^20^, ^21^, ^22^, ^23^, ^24^, ^25^ Among these, polymeric materials can easily be prepared in various shapes having desired physical properties, modified with different functional groups suitable for various biomedical applications such as drug delivery and tissue engineering.^26^ PLGA has been the most commonly used among polymeric NPs, because of its; (i) biocompatibility, (ii) approved status by both the United States Food and Drug Administration (FDA) and European Medicines Agency as a drug carrier, (iii) versatility for loading different types of drugs that may be water insoluble, and (iv) controllable biodegradation properties enabling tailored sustained release of drugs.^27^

Because of recent successes in biocoating of NPs, there is increased interest that has led to more effective drug delivery tools, and we review these recent developments. A wide range of cell types have been used as sources of these coatings leading to different patterns in biodistribution. There have also been several methods for preparing the coated NPs that we discuss. The biomedical applications of these NPs are potentially far reaching, and we critically analyze and discuss the preparation and use of biomimetic NPs, highlight limitations of the technology and present future directions that would advance this strategy for effective drug delivery.

## 2 CELL MEMBRANE CAMOUFLAGED PLGA NPs

### 2.1 Cell membranes

To achieve prolonged drug circulation, early research focused on the decoration of NPs' surface with synthetic polymers including polyethylene glycol (PEG), or encapsulating within liposomes or dendrimers to prevent their interaction with host environments.^28^ For decades, PEGylation has been the method of choice for stealth coating.^29^ However, recently it has been reported that PEGylated NPs are cleared from the circulation when second dose is administered because of an “accelerated blood clearance” phenomenon.^16^ Because of the antibody production against PEG, scientists have been exploring new avenues to deceive the body's immune system.^11^ Coating NPs with cell membranes is one of the most attractive approaches. This nature inspired strategy, leads to NPs that can efficiently interact with biological systems.^30^

Redesigning the functions and structures of the cells used for coating NPs can lead to engineered surfaces with improved control of the biological processes. Imitating cellular processes can increase our understanding of molecular interactions, and by engineering the surface, we improve these interfaces. The synthetic engineered systems could provide a simple model to study the pathways underlying disease occurrence/development. These insights will lay a foundation for improved diagnosis and treatment. Development of biomimetic systems that imitate cellular processes constitute a tremendous research tool and a promising therapeutic approach.^31^ In selecting cells as a source of lipid coating, consideration must be given to unique human proteins, antigens, that lack compatibility. The concepts used in transplantation biology including human‐to‐human transfer (allografts) and use of one's own cells (autograft) are relevant to coating NPs with cell membranes.

The cell membrane has a multitude of functions, one of which is protection from surrounding environment. The lipid bilayer that constitutes the major structure of the cell membrane contains a variety of proteins and carbohydrates that can be used as biomarkers, targeting ligands, or cloaking mechanisms. The molecules displayed on the cell surface impart specific functions and mechanisms of interaction with proximal cells and tissues. By wrapping synthetic NPs with the cell membrane, some of the functions are preserved and transferred to the NPs.^32^ Cell membranes can be derived from different cell types, such as red blood cells (RBCs),^33^ white blood cells (WBCs),^34^ platelets,^35^ stem cells,^36^ and cancer cells,^37^ and the characteristics of those cells can be transferred to the NP. The cell membrane disguises the particles, making them appear as “self” and thus not be rejected, or degraded, by the immune system. However, there are unique antigens on cell surfaces that can be immunogenic even if they are from human cells, for example, the Rhesus factor that is used in blood typing, and thus tissue matching remains relevant with these coated particles.^38^ Nonetheless, the newly inherited features of the membrane‐camouflaged NPs can be deployed for generating novel biointerfaces in the body that direct delivery as novel therapeutics.^39^

In thinking about directed delivery, as it pertains to cells, particles and cell‐particle hybrids, it is important to distinguish the mechanisms by which this can occur. The circulatory system distributes all materials in the blood around the body and the filtering organs such as liver and kidneys remove toxins, particulates and metabolic wastes and direct them toward excretion in the feces (via the liver) or the urine (via the kidneys). Circumventing the filtering process prolongs the circulation time and this is one objective for coating NPs. To direct an agent to a site of disease requires that the agent selectively accumulates at that site by sticking to or leaking into the target tissue. These mechanisms are possible with both particles and cells. Cells have the additional capability of sensing and actively migrating up a gradient, such as chemokine gradients; this active, energy‐dependent mechanism is homing. Particles cannot actively home to a target tissue; they can selectively bind, accumulate, leak or otherwise be retained at the target tissue. It is the non energy‐dependent characteristics of cell membranes such as the receptors they display, that may be transferred to NPs and optimizing these abilities will improve targeted delivery.

#### 2.1.1 RBC membrane‐based strategy

RBCs have a circulation time of 120 days in the blood, and this long circulation time has attracted attention for development of drug carriers with prolonged circulation. The unique shape (flexible biconcave) of RBCs enables them to pass through tiny capillary networks.^40^ The inherently non‐immunogenic, biocompatible and biodegradable nature of RBCs has led to them becoming a lead candidate for intravascular delivery. RBCs can form natural compartments which provide prolonged protection to the encapsulated drug in blood stream, and can also sustain long release rates for small molecules.^41^ A variety of proteins and glycans (carbohydrates) are displayed on the surface of RBC membranes that if transferred to NPs would help evade the immune system attack.^32^ One such type of integrin‐associated protein is cluster of differentiation (CD47) (often referred to as the “don't eat me” signal) that interfaces with its corresponding receptor on immune cells and enables RBC escape from destruction by circulating macrophages.^42^ Iron oxide NPs were coated with RBC membrane to escape immune clearance through interactions with the signal regulatory protein‐alpha (SIRP‐α) receptor. Immune cells express SIRP‐α which recognizes CD47 as a self‐signal and prevent endocytosis of RBCs by defense cells. The RBC membrane maintained the CD47 glycoprotein after transfer to NPs, and coated NPs utilized the CD47‐SIRP‐α interaction for prolonged circulation. It was demonstrated that RBC membrane is a better alternative to the current gold standard PEG for prolonging the systematic circulation time of NPs.^16^ However, RBCs lack active targeting capacity and related ligands, and thus additional functions must be added to direct delivery. Besides long circulating and selective targeting ability, controlled drug release is also required for an ideal drug carrier. In biomimetic NPs, the controlled release property can be conferred by the core materials.^24^

#### 2.1.2 WBC membrane‐based strategy

Leukocytes or WBCs are the population of blood cells comprised of monocytes, lymphocytes and granulocytes. These cells are prevalent in lymphatic and vasculature and can extravasate into extravascular space by amoeboid movement.^18^ Leukocytes are responsible for defense against infection and abnormal cells and are drivers of inflammation and antitumor immunity. During inflammation, selectins P and E are expressed on the surface of the endothelial tissue which facilitates binding of the leukocytes to the endothelium (blood vessel wall). The adherence of leukocytes to the endothelium can be used for targeting of drug carriers to sites of tissue damage or malignancy.^43^ Many of the key biological activities of WBCs are mediated by glycoproteins on the outer surface of the WBC membranes including CD47, lymphocyte function‐associated antigen‐1 (LFA‐1), and macrophage‐1 antigen (MAC‐1). Wrapping of NPs with WBC membranes transfers these cell surface markers to NPs, and some functions of the parent cells, such as site‐specificity and cellular self‐recognition, are retained.^44^ The surface proteins of WBCs imbue NPs with prolonged circulation, accumulation at sites of inflammation and disease, transendothelial migration and tumor tropism.^45^ Multifunctional WBC membrane‐based nanocarriers can be designed by employing the abilities native to leukocytes and they can be used for effective targeted delivery. These biomimetic NPs can be developed by either coating NPs with WBC membrane^46^ or with leukosomes (specific extracted surface proteins)^47^ or exosomes (leukocyte secreted extracellular vesicles) on core materials.^48^ WBC membranes confer both camouflages to protect from immune destruction and some targeting functions, and an understanding of the cell surface proteins is important for effective NP delivery.

#### 2.1.3 Platelet membrane‐based strategy

Platelets are small, nonnucleated cell fragments derived from megakaryocytes that circulate in the blood and are essential components for maintaining homeostasis. When blood vessels are injured, proteins such as collagen, which are present in the subendothelial layer, are exposed to platelets. After platelets bind to collagen, they release blood‐clotting factors for the purpose to stop bleeding and promote wound healing. This property of platelets can be utilized to target vascular injury sites by coating NPs with platelet membranes.^49^ Like RBCs, different proteins are expressed on the surface of platelet membranes such as CD47, CD55, and CD59, which can help to prevent the uptake of NPs by macrophages and can prevent unwanted immunological reactions.^50^, ^51^ The immune compatibility and prolonged blood residence time, owing to antigenic escape and both passive and active tumor target ability though binding to CD44 receptors upregulated on tumor surfaces, have been found to be useful when using NPs coated with platelet‐derived membranes as cancer theranostics.^45^ Platelet membrane‐coated NPs can also be applied for targeted delivery and enzyme responsive release of plasminogen activators for fibrinolytic therapy and to minimize off‐target systemic side‐effects. Plasminogen activators convert plasminogen to plasmin, which act to break down the fibrin mesh in formed clot, and it is used in the treatment of occlusive vascular conditions such as stroke and heart attack. Platelet membrane‐coated NPs were found to be capable of delivering their cargo to the clot within carotid artery in a thrombosis mouse model, without affecting the rest of the circulatory system.^52^

#### 2.1.4 Cancer cell membrane‐based strategy

Cancer cell membrane (CCM)‐cloaked NPs represent an ideal candidate for oncological applications. Cancer cells are robust and are readily cultured for membrane extraction. Unlike other cell‐derived membranes, CCMs may provide tumor‐targeting ability which can be exploited in the development of novel therapies for primary tumors and their metastases.^53^ The proliferation and metastases of cancer are primarily caused by advanced processes in which cancer cells escape the immune surveillance. CD47 molecule, which is overexpressed on CCM plays a key role in the process of immune eviction. CD47 molecule is particularly upregulated in some cancer cell lines such as MDA‐231, 4T1 and MCF‐7. Cell membranes rich in self‐biomarkers and self‐recognition proteins, can be isolated from cancer cells and wrapped around NPs, and enable them to evade the immune system in addition to providing tumor cell targeting.^32^ Besides CD47, other proteins that help in cancer cell self‐recognition and tumor self‐targeting are focal adhesion proteins, focal adhesion kinase, and RHO family proteins.^54^ Cancer cells can also express neoantigens, proteins unique to the cancer cell, and these may invoke an immune response that would lead to elimination of cloaked NPs from the circulation,^55^ serving as a cancer vaccine to redirect the immune system to the cancer cells, and thus have therapeutic benefit. CCM‐coated NPs can leak out passively through capillary network via enhanced permeability and retention (EPR) effect and can bind to tumor cells through homotypic identification. These biomimetic NPs display potential to target endothelium in vulnerable regions through heterotypic binding mechanisms, thus, adhesiveness of tumor cells confers the NPs ability to reach distant metastases.^56^

#### 2.1.5 Stem cell membrane‐based strategy

Mesenchymal stem cells (MSCs) are multipotent progenitor cells having the capability of both multi‐lineage differentiation and self‐renewal.^57^ The uniqueness of MSCs is attributable to the presence of surface molecules that are receptors for circulating ligands. These include receptors for cytokines, growth factors and chemokines,^58^ as well as proteins for cellular interactions and cell‐matrix adherence.^59^ Signaling pathways activated by receptor‐ligand interactions serve to connect the environment to cellular responses through expression of key metabolic and functional proteins. It has been reported that chemokines (CXCR1, CXCR2 CXCR4, CXCR5, CXCR6, CCR2, CCR7, CCR9, and CCR10) play a key role in homing, migration, and adhesion of MSCs to injured site or tumor.^60^, ^61^ In addition, MSCs possess ideal characteristics including non‐immunogenicity, long systemic circulation, and inflammatory/tumor‐specific properties.^62^ Certain stem cells such as MSC, hematopoietic stem cells, and neural stem cells can be exploited for drug delivery systems and carrying cargo to the tumor; these cells can secrete cytotoxic proteins that eliminate tumor cells.^11^ As an auspicious source of cell membranes, MSCs can be extracted from different tissues and expanded in the laboratory. Furthermore, MSC‐cloaked NPs can minimize blood clearance of circulating therapeutics by the reticuloendothelial system (RES) and increase cell specific uptake and enhance retention at tumor site. Although many of the functions present in MSCs do not transfer with the membranes, there are many passive interactions between the cell surface markers and the target tissues that are preserved.^63^ The design of biomimetic drug delivery systems by surface modification of functional NPs with MSC membranes has advanced in many areas,^64^ due to the unique properties of these cells.

Several cell types have been used as donors of cell membranes, and the key proteins that provide evasion and targeting properties are listed in Table 1.

**TABLE 1:** Properties and applications of cell membranes extracted from different cell types

Membrane source	Cell surface marker	Property	Application	Reference
RBCs	CD47	Immune evasion	Long circulation time, targeted drug delivery, antibacterial vaccine, treating autoimmune diseases, toxin removal	^42^, ^65^, ^66^, ^67^, ^68^, ^69^
Neutrophils	Integrin β2, Mac‐1, and LFA‐1	Selective targeting to inflammatory tissue	Inflammatory site targeting	^70^, ^71^, ^72^
Macrophages	CD126, CD130, CD120a/b, and CD119	Immune evasion, cytokine sequestration	Inflammatory site targeting, anti‐inflammatory	^73^, ^74^, ^75^
Platelets	CD47, CD55, and CD59	Specific targeting to injured tissue, binding to inflammatory Neutrophils	Targeting blood vessel injury, cancer metastasis targeting, treating atherosclerosis, treating autoimmune diseases	^50^, ^76^, ^77^, ^78^, ^79^
Stem cells	CXC, CC, and CX3C chemokine receptors	Tumor‐homing ability, movement across the endothelial lining	Tumor targeting, inflammatory migration	^58^
Cancer cells	CD47	Tumor targeting, antigen delivery	Homotypic targeting, cancer vaccine	^33^, ^37^

### 2.2 Poly(lactic‐*co*‐glycolic acid) NPs

Nanotechnology and polymer science have become the important areas of new developments in medical fields affecting a number of medical specialties and disease areas.^80^ Biodegradable polymers are commonly used in both pharmaceutical and medical fields.^81^ Among this group of polymers is PLGA, which is degraded mainly by hydrolysis, and traces of the breakdown products may be found in the urine.^82^ PLGA has favorable biocompatibility properties, and has thus been used for a wide range of applications such as tissue regeneration,^83^ osteofixation implants,^84^ and drug delivery.^85^, ^86^ PLGA is approved by FDA for use in several different applications, and it is the most commonly used biodegradable polymer for the preparation of NPs.^87^

#### 2.2.1 Characteristics of PLGA

PLGA has several adaptive features^85^, ^88^ and physical properties that make is a well‐suited carrier for drugs, proteins and other molecules including deoxyribonucleic acid (DNA) and ribonucleic acid (RNA).^89^ The degradation time of PLGA can be altered from days to years by changing the molar ratio of lactide and glycolide, which is a favorable property of PLGA. Due to their safety profile and sustained release property, PLGA‐based NPs are considered as effective nucleic acid delivery systems.^27^, ^90^ PLGA is prepared by the copolymerization of lactic acid and glycolic acid. The type and characteristics of PLGA are commonly specified by the molar ratio between these monomers. PLGA polymers having lactic acid and glycolic acid in a ratio of 50:50 is the most common type of PLGA used for biomedical applications. Glycolic acid monomer is less hydrophobic than the lactic acid monomer of the PLGA, thus increasing concentrations of lactic acid results in slower degradation and release rates. Copolymers in a 50:50 ratio have the fastest degradation and shortest shelf‐life compared to PLGA with ratios of 85:15 or 75:25.^91^, ^92^ The change in drug delivery properties and degradation rate of PLGA is dependent on the presence of functional carboxylic end groups that change its chemical structure. Covalent bonds formed between the carboxyl end‐groups of PLGA and amine groups of therapeutic agents, regulate drug release rates from PLGA nanodrugs.^93^ Block‐polymerization of PLGA and other copolymers can alter the physicochemical properties and behavior of PLGA. Block copolymers of PEG and PLGA are frequently reported in diblock (PEG‐PLGA)^94^ or triblock conformations (PEG‐PLGA‐PEG or PLGA‐PEG‐PLGA).^95^, ^96^ The PEG coating can enhance the shelf stability by reducing interactions with foreign molecules. However, it can also decrease the encapsulation efficiencies of drugs. Diblock polymers have shown improved release kinetics than PLGA alone.^89^ The random polymerization of PLGA with other polymers will be beneficial; for example, combining PLGA with biodegradable photoluminescent polyester will make the system suitable for photoluminescence imaging.^97^ The physical properties of PLGA nanostructure can be controlled by parameters specific to the preparation method employed. For example, concentration of PLGA used for preparation of NPs can determine the size of PLGA NPs to a certain extent. Surface decoration is another parameters that allows a certain control over particles' biodegradation, biocompatibility, blood half‐life and, when applicable, targeting efficiency.^98^ PLGA can be dissolved in wide range of organic solvent, depending on lactic acid and glycolic acid composition. If the ratio of lactic acid is high, the PLGA will dissolve by chlorinated solvents, such as chloroform or dichloromethane, and by water‐miscible solvents, like tetrahydrofuran or acetone. While, if ratio of glycolic acid is high, it will dissolve by fluorinated solvent, like hexafluoroisopropanol.^99^ Finally, the glass transition temperature of PLGA can is above body temperature, between 40 and 60°C and this decrease with a decrease in the molecular weight.^100^

Degradation of PLGA occurs in the following steps:

- Hydration: Aqueous environment causes heterogeneous or bulk erosion of the PLGA. Penetration of water occurs in the amorphous area of the polymer and hence hydrogen bonding and van der Waals forces are broken, and the glass transition temperature is lowered.
- Initial degradation: Initial polymer degradation starts after the breakage of the covalent bonds.
- Continuous degradation: The degradation process is auto‐catalyzed by carboxylic end groups of PLGA due to continuous hydrolytic reactions. Disruption of the covalent bonds results in mass loss which further causes loss of the structural integrity.
- Dissolution: Oligomers and monomers of lactic acid and glycolic acid are produced as end products. Both monomers are substrates for the Krebs cycle and are excreted from body in the form of water and carbon dioxide.^92^, ^93^, ^101^

#### 2.2.2 PLGA‐based NPs as drug carriers

PLGA possesses the capability to load both water soluble and insoluble drugs and can be used to deliver these drugs to improve the management of disease. PLGA‐based NPs have been widely explored for specific delivery of drugs to target sites in cancer therapy. PLGA‐based nanodrugs offer the benefits of active or passive targeting for delivery to the tumor site, and limiting the exposure of surrounding healthy tissues to the drug.^102^ For the treatment of neurological diseases, the brain by blood–brain barrier (BBB) limits access, and polymer based NPs have been extensively explored to improve delivery across the BBB.^103^, ^104^ These successes have led to development of PLGA‐based therapeutics for a number of diseases.

In cardiovascular diseases (CVDs), nanostructured drug delivery systems have largely been based on functionalized PLGA.^105^ Blood vessel restenosis is a problem that occurs following endovascular procedures and has presented challenges for therapy. There are various drugs such as immunomodulators (cyclosporine‐A, steroids) and smooth muscle cell inhibitors (doxorubicin [DOX], paclitaxel) that are used in the management of the restenosis, but sustained release at sites of tissue damage is desirable to improve therapy. Controlled release of these drugs and protection from degradation, have been based on loading these molecules onto PLGA NPs. Selective inhibitors of platelet‐derived growth factor receptor and protein tyrosine kinase (AG‐1295 and AGL‐2043) have also been loaded onto PLGA NPs, and these were shown to prevent restenosis of balloon injured carotid artery in rats.^106^ Despite these advances, there remains a need for sustained release over long durations and this may be possible with further functionalization of the NP surface.

Anti‐inflammatory drugs have also been delivered with PLGA NPs. For management of joint inflammation, superparamagnetic iron oxide (SPIO) containing PLGA NPs have been used for the prevention of articular inflammation. SPIO NPs and the corticosteroid drug dexamethasone acetate were co‐encapsulated into PLGA microparticles in order to locally treat arthritis.^107^ Anti‐inflammatory drugs are known for causing problems if taken orally and have been shown to cause or exacerbate inflammatory bowel disease (IBD); use of parenterally administered drug nanocarriers can reduce or eliminate these GI problems. The size of the particles enables them to accumulate in the inflamed cartilage, and with controlled release of anti‐inflammatory agents, the symptoms can be controlled. PLGA‐based NPs have demonstrated control of other chronic inflammatory diseases including in the management of IBD, where NPs have shown to accumulate in the inflamed mucosa.^108^ Demonstrating accumulation of NPs in the target tissue can be enabled by incorporating distinct molecular signatures into the nanocarriers that can be detected by various imaging modalities. This combination of imaging and therapy in one NP has been referred to as theranostics.

Theranostic agents are a combination of therapeutics and diagnostics in single multifunctional formulations, and a vast majority of these agents are nanoparticulate carrier systems given the carrying capacity and versatility of NPs for functionalization.^108^ An example of the flexibility in design of NPs are core–shell PLGA particles containing gold NPs and dye within the shell, and perfluorohexane liquid in the core.^109^ These NPs were administered to rabbits with metastatic squamous carcinoma, and upon laser irradiation, the liquid core was activated, resulting in damage to surrounding cancer cells and necrotic regions of the tumor.^109^ Another example of a theranostic NPs is dual SPIO‐paclitaxel (PTX)/loaded PLGA NPs. Relaxometry and magnetic resonance imaging (MRI) demonstrated that these NPs were T_2_ contrast agents, and PTX was loaded into these NPs as the cytotoxic agent. Drug loaded NPs were cytotoxic in culture and controlled tumor growth in vivo, while SPIOs alone showed no toxic effect in CT‐26 cells and no efficacy in vivo. These examples demonstrate the multifunctional nature of nanodrugs and the ability to direct and monitor the delivery of drugs to target tissues; this will enable simultaneous molecular imaging, drug delivery and real‐time monitoring of the therapeutic response.^110^ Further functionalization of these theranostics with cell membrane coatings offers additional opportunities for delivering and monitoring therapy.

### 2.3 Combination of cell membrane and PLGA NPs

Cell membrane‐camouflage technology dates to 2011, when a landmark paper in which the cell membrane was extracted from erythrocytes and used for coating PLGA NPs in a top‐down approach was published. Injection of the resulting particles into mice revealed longer circulation time of cell membrane‐cloaked NPs as compared to PEGylated NPs. The biodistribution study showed blood retention time for erythrocyte membrane‐coated NPs of up to 72 h.^13^ The field has been building on this pioneering work with erythrocyte membranes, by using other cell membranes to produce multifunctional membrane‐coated PLGA NPs.

Building on the theme, Hu et al. reported the use of platelet‐derived membrane‐coated PLGA NPs.^51^ In this study, platelets were selected because they have the distinctive property of sticking to surfaces of diseased or injured tissues. The resulting biomimetic NPs displayed platelet‐imitating characteristics including selective binding to injured blood vessels of rodents and humans, increased binding to platelets with adherent pathogens.^51^ An example of another cell type was neutrophil membrane‐coated PLGA NPs.^111^ With these NPs, it was possible to overcome the blood–pancreas barrier and achieve site‐specific drug delivery. It has been postulated that homotypic cells (i.e., cells matching the disease) migrate to sites of the disease, and this was demonstrated using membranes from a homotypic HepG2 hepatocellular carcinoma (HCC) cell to direct chemotherapy to liver cancer (HCC).^112^ Cell membranes may also facilitate uptake by resident cells in the diseased tissues as an additional mechanism for targeting. Human umbilical cord‐derived MSC membrane‐coated DOX containing PLGA NPs were fabricated using ultrasonication.^113^ The in vitro uptake of membrane‐cloaked NPs by cancer cells was threefold higher than that of bare PLGA NPs, leading to enhanced cancer cell cytotoxicity.

While cell membrane cloaking provides notable stealth‐ability, additional selective targeting may further reduce their side effects and increase their therapeutic effect. The cell membrane‐camouflaging approach has been progressively applied to more and more complicated biological systems using more varied and sophisticated engineering to incorporate a greater diversity of ligands and enhance their performance. The coated cell membrane can be directly modified using strategies such as covalent modification, non‐covalent modification or enzyme‐involved modification.^114^ In covalent modification, amide bonds are formed between amine groups on the surface of the cell membrane and carboxylic acid groups present in the therapeutic moiety.^115^ In non‐covalent modification, lipid insertion is a commonly used, simple and stable approach. In this method, the functional group, related to lipids, can be spontaneously inserted into the phospholipid bilayer via hydrophobic interactions. If the functional moieties are linked with multiple hydrophobic interactions, then higher binding force is required. In contrary, the lipids insinuated into the exterior of cell membranes show good stability and strong molecular adherence to the target cells.^116^ Along with lipid insertion, binding of certain peptides and antigens to the membrane surface proteins of cell membrane‐coated NPs via ionic bond and hydrophobic interactions is another non‐covalent modification strategy. However, this conjugation generally has a random distribution and the conjugated protein may lose function when changes to functional domains occur.^117^, ^118^ In enzyme‐based modification, the therapeutic moiety is introduced onto the cell membrane via an enzymatic reaction, yielding high selectivity. For example, the enzyme, glycosyltransferase, can be used to introduce the therapeutic moiety CD44, derived from human MSCs, which selectively binds to P and E selectins on target cells.^119^ Enzyme‐involved reaction is also a kind of covalent modification. This approach has obtained a promising progress in cell membrane modification. But, it is difficult to utilize it to construct membrane‐engineered cell membrane‐coated NPs as each reaction demands the unique enzyme, which is challenging to be separated and purified from living cells. Also, it is hard to fully control the biochemistry reaction rate, which can be affected by different parameters, such as reaction temperature and membrane proteins.^120^ Genetic engineering is another sensible strategy used for cell membrane's modification. In general, transmembrane proteins (TMPs) share two necessary compartment of cell membrane anchoring: the hydrophobic peptide region and cleavable N‐terminal signal sequence. Genetic engineering needs a solid knowledge of trans‐membrane site sequences, location of signal peptide sequence and topological domains to native TMPs. This information will help in integration of desired amino acid sequences into desired distinction sites of targeted proteins via gene editing methods.^121^ For example, Kell and glycophorin A are membrane proteins with an exposed N and C terminus on the mature erythrocytes with a single trans‐membrane region. Utilizing this structural information, the LPXTG motif for the sortase A reaction can be inserted in outer leaflet of cell membrane in vitro using genetic engineering method. These artificially expressed motifs could maintain their property and introduce a single‐domain antibody attached to the cell membrane with a sortase A reaction.^122^ Adoptive T lymphocytes transfer is a promising approach for cancer immunotherapy having genetically modified chimeric antigen receptor (CAR). Such CAR T cells when targeted to tumor surface, recognize the surface antigens independently and kill the tumor cells upon antigen contact.^123^ After transduction by retrovirus, CAR‐T cells expressing antigen receptors can provide genetically engineered membranes to coat the inner cores. These novel camouflaged nanocarrier combined the targeting ability of CAR‐T cells with the advantages of nanoscale cores.^124^

#### 2.3.1 Methods of preparation of PLGA‐based cell membrane‐camouflaged NPs

**FIGURE 1:**
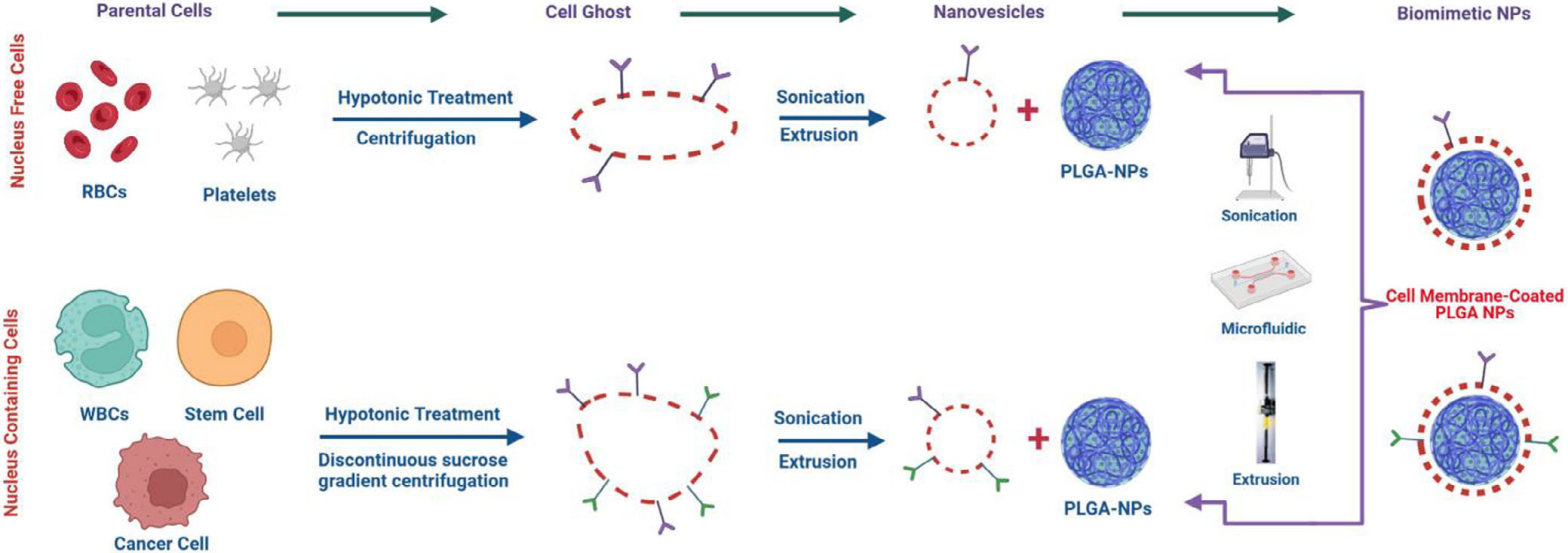
Schematic illustration of preparation of cell membrane‐coated poly(lactic‐*co*‐glycolic acid) (PLGA) nanoparticles (NPs). Created with BioRender.com.

The fabrication of cell membrane‐camouflaged NPs requires cell membrane extraction from the parent cells, fabrication of PLGA NPs, and coating of NPs using extracted cell membranes (Figure 1). There are several published methods and approaches that are described below.

##### Extraction of cell membrane

Extraction of the plasma membrane from cells must be gentle to limit dissociation and denaturation of the membrane‐associated proteins. Cell membrane isolation typically includes a mild cell lysis followed by isolation of cell membranes, and varies according to cell type, specifically with respect to nucleated and nonnucleated cells.

###### Membrane extraction from nonnucleated cells

Maturation of both RBCs and platelets includes nonnucleation, and the resulting nonnucleated cells are the most common nonnucleated cells in human body.^125^ To extract the bioactive plasma membranes from nonnucleated cells, the cells are first isolated by blood fractionation via centrifugation, the recovered cells are then lysed with either repeated cycles of freeze–thaw or treatment with hypotonic solutions. Another centrifugation step serves to remove the soluble proteins, leaving the vesicles in the pellet. The purified vesicles are then passed through nanosized pores containing polycarbonate membranes to generate nanovesicular structures. The samples are usually stored at 4°C in the presence of protease inhibitors to maintain the integrity and bioactivity of the membrane and associated proteins.^126^

###### Membrane extraction from nucleus‐containing cells

The cell membrane extraction from leukocytes, cancer and stem cells is slightly more complicated than that of nonnucleated cells. Parent cells are harvested from blood samples (WBC) or culture dishes (stem cells or cancer cells) and isolated by centrifugation. The homogenization of cells is via sonication to disrupt the cells, and the plasma membranes are separated from nuclei in the mixture by high‐speed gradient centrifugation. The membrane rich portion is again washed with isotonic buffers to get membrane vesicles, which are then sonicated and passed through polycarbonate membrane to obtain nanovesicles.^127^

##### PLGA NP core

Several techniques are available for the synthesis of PLGA NPs which can be subdivided into two categories, that is, top‐down and bottom‐up techniques. Top‐down approach utilizes a preformed polymer to produce the NPs. Top‐down approaches for the synthesis of PLGA NPs include emulsion diffusion, emulsion evaporation, salting out, and solvent displacement processes. Bottom‐up approaches employ the monomer as the starting point for the synthesis of NPs and the techniques of emulsion polymerization, precipitation polymerization and interfacial polymerization are used.^128^ The most widely employed techniques are described in more detail in the next section.

###### Single emulsion method

The single emulsion technique is the most frequently used approach for the synthesis of PLGA NPs. Oil in water (O/W) emulsification is mostly employed when the drug to be encapsulated is hydrophobic or poorly soluble in water. The organic phase is prepared by dissolving required amount of polymer in a specific volume of organic solvent like chloroform, dichloromethane or ethyl acetate, followed by the addition of drug into this solution, and as a result, dispersion is formed. Under constant stirring, this dispersion of drug and polymer is added into aqueous phase containing suitable surfactants such as polysorbate 80, poloxamer 188, and polyvinyl alcohol leading to the formation of a stable emulsion. The volatile organic solvent is evaporated either by continuous magnetic stirring or by using rotary evaporator at reduced pressure.^129^

###### Double emulsion method

The double emulsion method is known as water in oil in water (W/O/W) technique. The particle size and drug entrapment are primarily influenced by the type of organic solvent and stirring speed. During this procedure, the desired amount of drug is dissolved in water, and then added into an organic phase comprised of PLGA dissolved in suitable organic solvents under continuous vigorous stirring. This results in the development of W/O primary emulsion. The process of emulsification is continued by the addition the primary emulsion into an aqueous solution, which is maintained by stirring while the organic solvent evaporates.^130^

###### Emulsion diffusion method

Organic solvent (e.g., ethyl acetate, acetonitrile, benzyl alcohol, etc.) is used for dissolving the synthetic polymer (PLGA); PLGA has partial miscibility with water. This step leads to the formation of an organic phase. The emulsification of the organic phase is carried out with water containing suitable emulsifier (i.e., nonionic polyvinyl alcohol, anionic sodium dodecyl sulfate, or cationic didodecyldimethylammonium bromide) with continuous stirring. The formation of polymeric NPs is induced by the counter diffusion of water and diffusion of the organic solvent into the emulsion droplets. The key parameters that influence the size of the NPs produced by emulsion diffusion are; copolymer ratio, solvent nature, amount of polymer, viscosity, molecular mass of polymer and surfactant, phase ratios, temperature, stirring speed, and flow rate of added water.^131^

###### Nanoprecipitation method

The nanoprecipitation method used for fabrication of polymeric NPs is a one‐step process which is also recognized as the solvent displacement technique. A system containing three basic parts; the polymer, the polymer solvent and the nonsolvent of the polymer is utilized in the nanoprecipitation technique. This technique is frequently employed for the entrapment of water insoluble drugs, but it is suitable for water soluble drugs as well. Drugs and polymers are dissolved in water‐miscible polar solvent such as acetonitrile, acetone, methanol or ethanol. The solution is then added into an aqueous solution in a controlled manner containing a suitable surfactant. Rapid solvent diffusion is used immediately for the development of NPs. Finally, the organic solvent is rotary evaporated under reduced pressure.^102^

###### Salting out method

In this method, a water‐miscible organic solvent like tetrahydrofuran or acetone is used for dissolving the polymer, which leads to the formation of organic phase. This organic phase is then emulsified in an aqueous phase, under continuous stirring. The aqueous phase consists of high salt and emulsifier concentrations which are immiscible in the water. Particularly, the salts such as magnesium acetate tetrahydrate or magnesium chloride hexahydrate are used as 60% (w/w) in a salt to polymer ratio of 3:1. Unlike the emulsion diffusion method, here, the diffusion of solvent does not occur in the presence of salts. Rapid addition of water into the o/w emulsion, under gentle stirring, decreases the ionic strength and results in passage of the water miscible organic solvent into the water phase, which leads to the formation of nanospheres.^128^ Purification is the last step and can be performed by centrifugation or cross flow filtration to eliminate the salting out agent. Electrolytes such as (sodium chloride, magnesium chloride, or magnesium acetate) or nonelectrolytes like sucrose are the frequently used as salting out agents.^132^

##### Fusion of NPs and membrane vesicles

###### Extrusion

In extrusion process, the membrane vesicles and NP cores are continuously extruded through polycarbonate membranes of different pore diameters at least five times to get the particles of required size. Excess of cell membrane is usually used to completely wrap the NPs. This method is mostly used for coating polymer‐based NPs with the size range of 65–340 nm. Camouflaged NPs fabricated in this way can be used to encapsulate drugs more efficiently, and they show uniform size distribution. However, this technique is laborious and not economical for use in industrial scale.^75^

###### Sonication

In this process, the electrostatic interactions cause the NP cores to fuse with plasma membranes. Wei et al. prepared platelet membrane enclosed PLGA NPs through sonication employing a bath sonicator at 42 kHz frequency and 100 W power for 2 min.^35^ This is a simple procedure and leads to polydisperse preparations. However, for optimum fusion capacity and prevention of membrane surface protein denaturation, the ultrasonic frequency, power, and duration should be optimized.^9^ The sonication method can spontaneously produce core–shell NPs with reduced destruction of cell membrane structure. However, sonication is not appropriate for the production of cell membrane‐coated NPs in large quantities.^133^

###### Combined sonication and extrusion

The combination of sonication and extrusion was used for fabrication of platelet membrane wrapped chitosan oligosaccharide‐PLGA NPs containing bufalin. In this method, the NPs and platelet membrane are first mixed and then sonicated at 40 kHz frequency and 100 W power for 5 min. This mixture is then repeatedly passed through a mini extruder with 200 nm sized pore membrane to get the final camouflaged NPs.^134^

###### Microfluidic sonication

In microfluidic sonication, a microfluidic device with two stages is immersed into bath sonicator before starting the fabrication process of membrane‐cloaked PLGA NPs. The first stage of the device consists of three inlets and one straight microchannel, while the second stage contained one inlet, one outlet and one double‐spiral microchannel. In one example, preparation of CCM‐coated PLGA NP, a solution of PLGA in dimethylformamide and trifluoroethanol (7 ml/h), two 0.15 mg/ml solutions of CCM in PBS (80 ml/h each), and a PBS solution (7 ml/h) were introduced from the center and two inlets at the first stage and the inlet at the second stage.^135^ Strong micro vortices are generated in the main microfluidic channel inside the spiral geometry and around the inlets. Under a specific flow rate, the four micro vortices vertical to the flow direction at the junction of the inlets provided a chaotic mixing of PLGA solution with PBS, leading to rapid and an efficient interfacial precipitation of PLGA NPs. By applying sonication, CCM‐PLGA core−shell NPs were prepared. Compared to other strategies, the use of microfluidic systems in has obvious advantages in reducing membrane surface protein loss and maintaining membrane integrity.^136^

## 3 BIOMEDICAL APPLICATIONS OF CELL MEMBRANE CAMOUFLAGED PLGA NPs

PLGA‐based biomimetics have been utilized for the fabrication of drug delivery systems to enhance the efficacy of NPs in treating a wide range of diseases. Applications of different cell membrane‐coated PLGA NPs, experimental models and the outcomes of the studies are summarized in Table 3. The primary objectives of these modifications are to improve delivery to the target, increase circulation time for a more uniform delivery, and to reduce the systemic toxicity associated with conventional nanoparticulate drug delivery.

### 3.1 Cancer therapy

Cancer treatment options include a wide variety of approaches and often include a combination of chemotherapy, surgery, radiotherapy, phototherapy, and immunotherapy with advances being made in combinatorial strategies as hybrid technologies.^137^, ^138^ Yet, there is no panacea and the limitations of conventional methods related to therapeutic performance, tumor targeting specificity and off‐target toxicities are being addressed by modification of nanosized drug delivery systems. Because these nanocarriers offer improved biosafety, performance, and bioavailability of a wide variety of therapeutic agents, they are defining the next generation of cancer therapies.^139^ Biomimetic NPs with PLGA at their core can carry cytotoxic anticancer agents, or photoactivatable near‐infrared dyes used in photodynamic therapy to the tumor, and are beginning to offer options to the oncologist for treating cancer patients.^140^, ^141^ Several of these are discussed below.

#### 3.1.1 RBC membrane‐coated NPs

**FIGURE 2:**
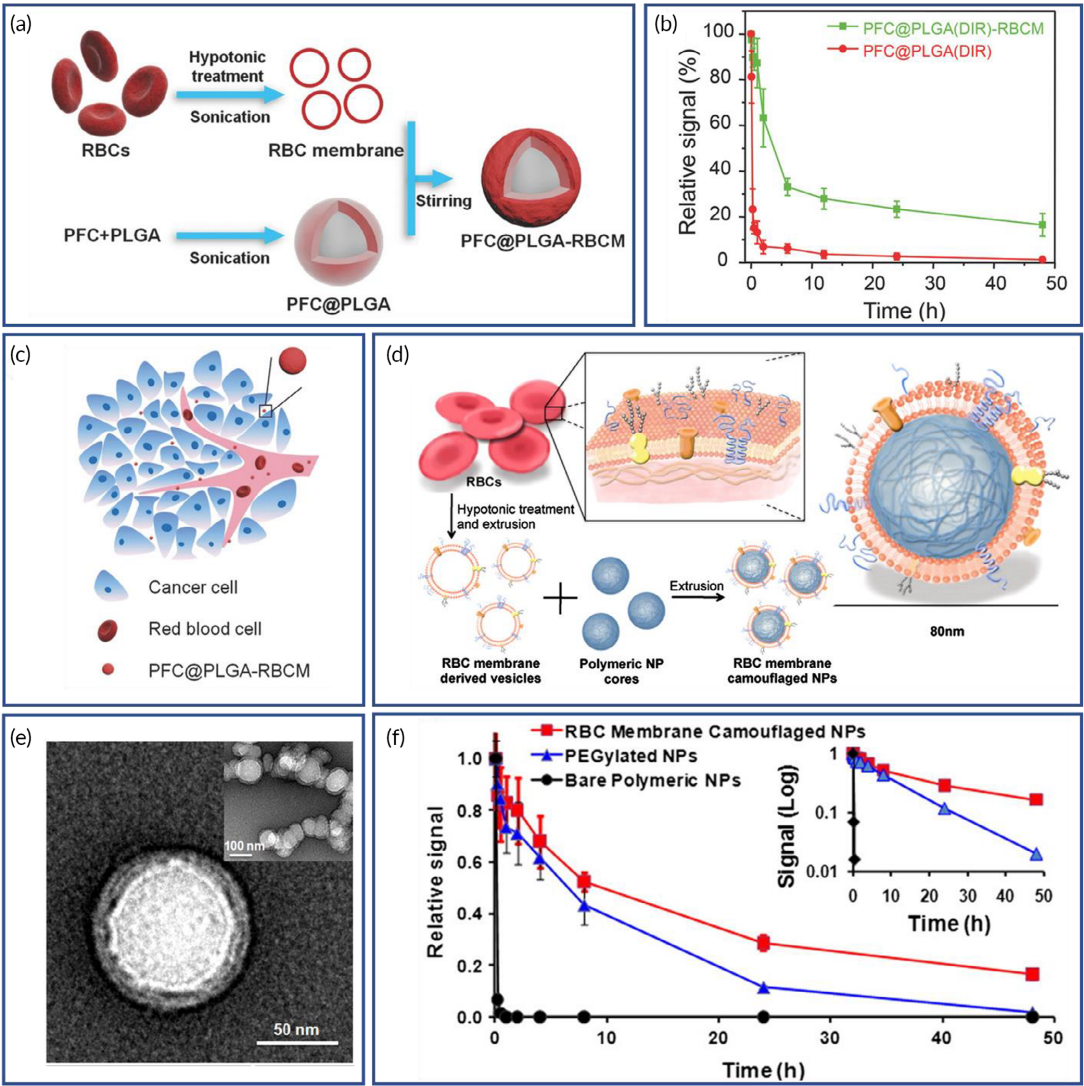
Red blood cell (RBC) membrane‐coated nanoparticles (NPs) for the treatment of hypoxia and cancer radiotherapy (a–c) and verifying the effect of coating on the duration of cargo circulation (d–f). (a) Schematics of the preparation of RBC membrane‐coated poly(lactic‐*co*‐glycolic acid) (PLGA) NPs encapsulating perfluorocarbon (PFC@PLGA‐RBCM). PFC solution was encapsulated inside the PLGA shell, which was then coated with RBCM. (b) Blood‐circulation curves of PFC@PLGA and PFC@PLGA‐RBCM in nude mice at different time points post injection. (c) Schematics of PFC@PLGA‐RBCM nanoparticles penetrating inside solid tumors through blood vessels. Reproduced with permission.^142^ Copyright 2017, Wiley. (d) Schematics of the preparation process of the RBC membrane‐coated PLGA nanoparticles. (e) TEM image of the nanoparticles. (f) Systemic circulation lifetime of the intravenously injected nanoparticles. Inset shows the change in the pharmacokinetic behaviors where slope of the plots can provide the circulation half‐life. Reproduced with permission.^13^ Copyright 2011, Proceedings of the National Academy of Sciences of the United States of America.

RBC membrane‐cloaked PLGA NPs containing perfluorocarbons were produced for the treatment of hypoxia and enhancing cancer radiotherapy (Figures 2a,c).^142^ The perfluorocarbon loaded PLGA NPs coated with RBC membrane showed efficient loading of oxygen with enhanced circulation in the bloodstream (Figure 2b). Due in part to the “don't eat me” signal on the RBCs; without CD47 on the surface, the RBCs are rapidly cleared from the blood circulation by macrophages.^42^ These NPs significantly improved extravascular diffusion within the tumor, providing remarkably enhanced treatment efficacy during radiotherapy. In order to evaluate the immunogenicity, in vitro cytotoxicity, cellular uptake, in vivo therapeutic efficacy and safety of RBC membrane coating, DOX‐loaded RBC membrane‐coated PLGA NPs were developed and tested in a murine lymphoma model.^143^ DOX loaded RBC membrane‐PLGA NPs had enhanced biocompatibility and immune evasion, and DOX loading enhanced cellular toxicity as compared to free DOX in EL4 lymphoma cells, and improved efficacy in terms of inhibiting tumor growth compared to the use of free DOX when tested in the mouse model.^143^

RBC membrane‐cloaked PLGA NPs were fabricated to check whether the coating increases the duration of circulating cargo (Figure 2d).^13^ NPs had a polymer core (~70 nm in diameter) and a lipid shell (~7–8 nm in thickness), confirming the membrane coating on the polymer particle surface (Figure 2e). Protein characterization studies indicated that during the particle synthesis, the composition of membrane proteins was preserved with key markers still detectable on the RBC membrane cloaked PLGA NPs. In vivo pharmacokinetics studies showed that RBC membrane‐coated NPs had a prolonged half‐life in the circulation, indicating their superior retention in blood compared to conventional PEGylated NPs (Figure 2f). Elsewhere, curcumin and tirapazamine were loaded into RBC membrane‐cloaked PLGA NPs for improved treatment of hypoxic tumors with chemotherapy^144^; the hypoxic tumor microenvironment typically requires multiple dosing to achieve therapeutic efficacy. Because of the natural membrane coating, the NPs in this study did not provoke any notable hemolytic or immunogenic reaction, validating the biocompatibility.^144^ The erythrocyte membrane‐coated NPs in this study showed excellent cytotoxicity for A375 and MCF7 cancer cells in 2D cultures and 3D spheroids. The decrease in the migration and down regulation of mesenchymal markers makes this curcumin delivery method an ideal candidate for chemotherapy, and tirapazamine is designed for treating hypoxic tumors.^144^ RBC membrane‐cloaked tetrandrine containing PLGA NPs were also reported to be capable of reversing multidrug resistance (MDR) in tumors^145^; tetrandrine has great potential in reversing MDR in cancer therapy. The MDR reversal was significant when the NPs were administered in combination with Adriamycin to drug‐resistant mammary carcinoma cells. Pharmacokinetics studies showed that the free drug was quickly cleared from the circulation, and that the half‐life and mean residence time of the erythrocyte membrane‐coated tetrandrine‐loaded PLGA NP group were longer than those of the free drug.^145^

#### 3.1.2 WBC membrane‐coated NPs

**FIGURE 3:**
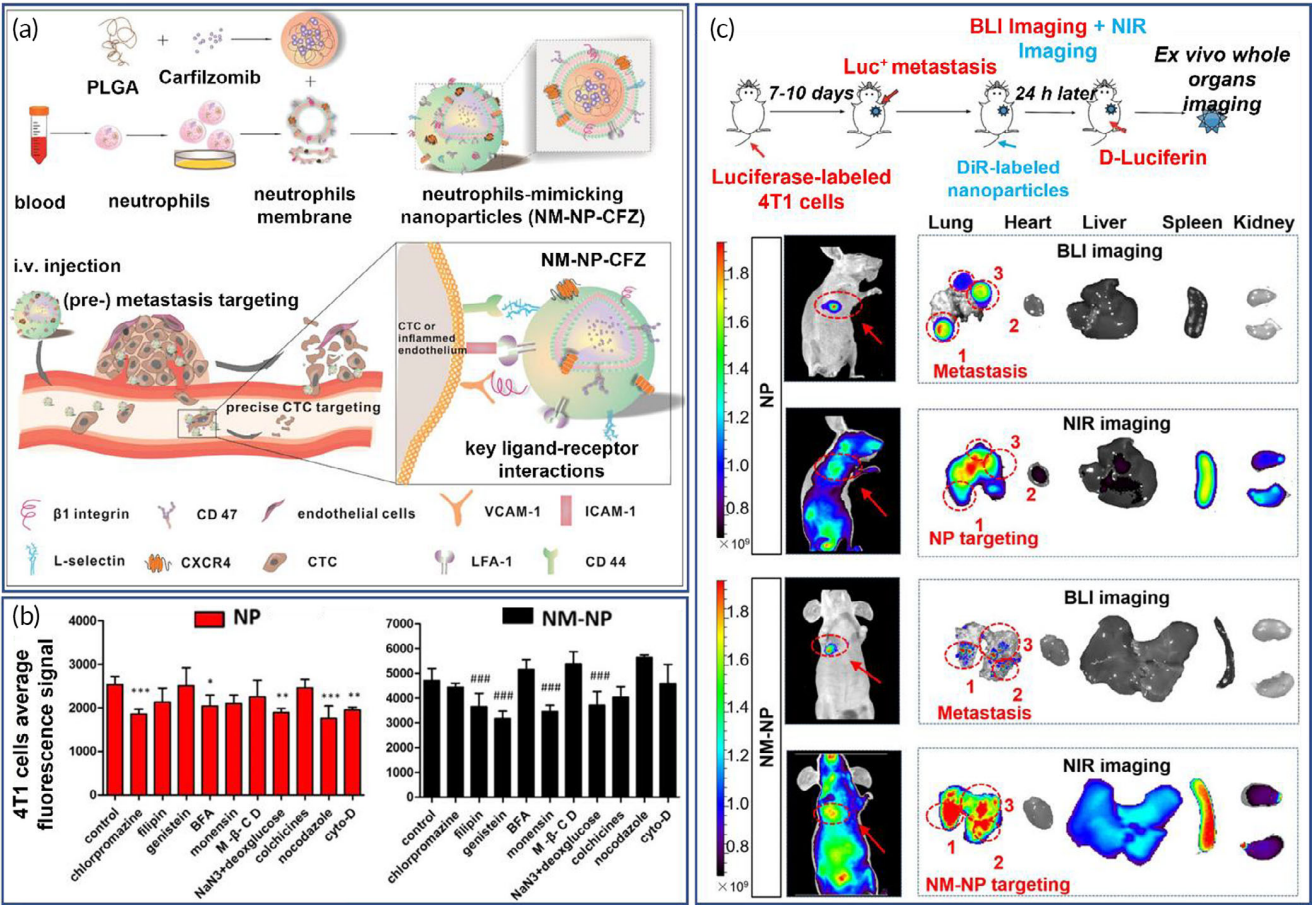
Neutrophil membrane‐coated nanoparticles (NPs) for the delivery of carfilzomib to treat metastatic cancer. (a) Schematic illustration of neutrophil‐coated poly(lactic‐*co*‐glycolic acid) (PLGA) NPs loaded with carfilzomib. (b) Quantitative analysis of cellular association of coumarin‐six‐labeled NPs and NM‐NPs in 4T1 cells following pre‐incubation with various endocytosis inhibitors. (c) Dual‐mode imaging of mice with metastasis 14 days after luciferase‐labeled 4T1 cells injection in vivo and ex vivo. Reproduced with permission.^146^ Copyright 2017, The American Chemical Society.

PLGA NPs were coated with neutrophil membranes for the delivery of carfilzomib to treat metastatic cancer (Figure 3a).^146^ The endocytosis pathways study indicated that neutrophil membrane‐coated NPs were internalized with the involvement of lysosome as opposed the bare NPs which were internalized via the involvement of Golgi apparatus. It was concluded that neutrophil membrane changes the endocytosis pattern (Figure 3b). Besides, the coated NPs showed capability of binding to circulating tumor cells, and homing to the pre‐metastatic endothelium, and in vivo, they had improved cellular targeting capability and increased accumulation in the pre‐metastatic niche (Figure 3c).^146^ Zhang et al. synthesized cytotoxic T‐lymphocyte membrane wrapped paclitaxel loaded PLGA NPs and tested these in combination with low dose irradiation.^147^ In immune deficient mice, the human T‐lymphocyte membrane‐coated NPs inhibited the growth of human gastric cancer by 56.68%. Moreover, the administration of low dose irradiation at the tumor site led to reduced tumor growth rate (88.50%), overexpressed adhesion molecules in the tumor vessels and contributed to the retention of T‐lymphocyte membrane‐PLGA NPs at the target site.^147^ Another study reported coating of mPEG‐PLGA NPs with natural killer (NK) plasma membranes to achieve effective obstruction of primary and abscopal (i.e., distant from the irradiated site) tumor growth in mice.^148^ The NK cell membrane‐coated NPs showed enhanced immunogenic responses following photodynamic therapy. Proteomic profiling of NK plasma membrane showed targeting effects at the tumor site and initiated M1 macrophage polarization. Moreover, NK cell membrane‐coated NPs mediate the immunogenic response that could increase immunotherapy and produce increase antitumor immunity. These treatments led to eradication of the primary tumor, and inhibition of early distal metastases.^148^

#### 3.1.3 CCM‐coated NPs

**FIGURE 4:**
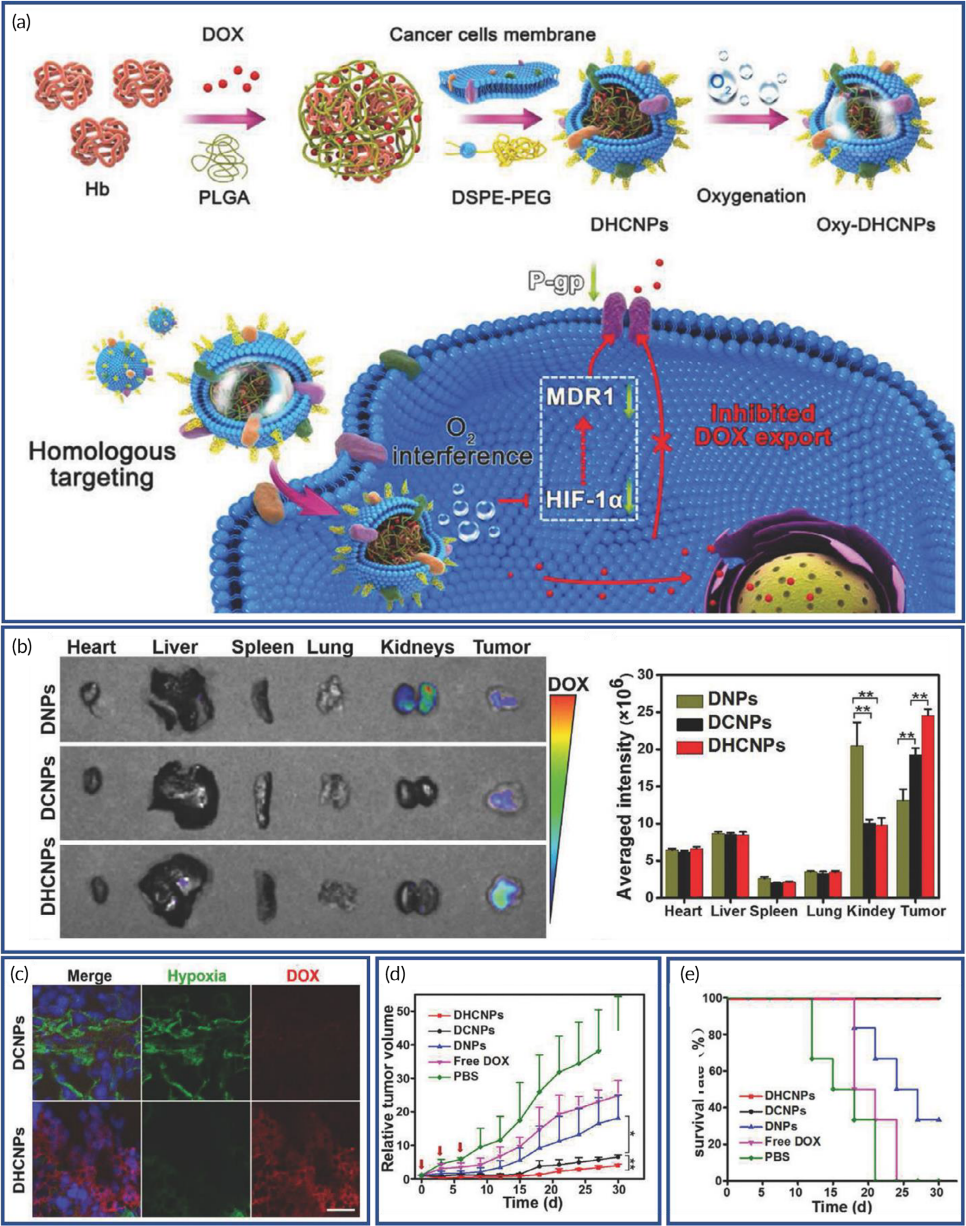
Cancer cell membrane‐coated nanoparticles (NPs) for the delivery of oxygen to tumor site. (a) Schematic representation of fabrication of cancer cell membrane‐cloaked poly(lactic‐*co*‐glycolic acid) (PLGA) NPs loaded with doxorubicin/hemoglobin (DOX/Hb) (DHCNPs). (b) Ex vivo fluorescence images of major organs and tumors of nude mice after 24 h preinjection of DOX loaded PLGA‐lipid NPs (DNPs), DOX loaded PLGA‐cancer cell membrane NPs (DCNPs), and DOX/Hb loaded PLGA‐cancer cell membrane NPs (DHCNPs) and semiquantitative biodistribution of DNPs, DCNPs, and DHCNPs in nude mice. (c) Fluorescent imaging of hypoxic probe (green) and DOX (red) in tumor sections at 24 h after intravenous injection of DHCNPs (scale bar 25 μm). (d) MCF‐7 tumor growth curves of different groups (scale bar 50 μm). (e) Survival rates of tumor‐bearing mice in various groups. Reproduced with permission.^150^ Copyright 2017, John Wiley & Sons.

CCM coating is also widely used for cancer targeting due to its propensity for homologous adhesion. Homotypic adhesion is a process in which cancer cells stick to each other and facilitate tumor growth.^149^ In one study, the use of homotypic CCM‐coated HepM‐PLGA DOX nanocarriers showed enhanced drug delivery and efficacy in HCC.^112^ HepM‐PLGA particles delivered DOX directly to the tumor site in mice with a resultant decreased tumor volume (by ~90%). The use of biomimetic homotypic CCM was demonstrated in the treatment of HCC, and it is suggested as a versatile approach for the treatment of other cancers. Another strategy for treating hypoxia‐induced chemoresistant cancers, hemoglobin (Hb), and DOX containing CCM‐coated PLGA particles were developed.^150^ Hb and DOX were encapsulated in PLGA, forming the core, in which the CCM and PEGylated phospholipid were covered to build the DHCNPs (Figure 4a). The accumulation of NPs in tumor and major organs was evaluated, where lower amount of DOX was observed for the coated NPs which were explained by the escape from renal clearance due to the CCM serving as a camouflage. Besides, DOX accumulation in tumor was considerably increased for the coated NPs compared to the control groups (Figure 4b).

Treatment with DCNPs without O_2_ supply displayed large‐scale hypoxia distribution in tumor and low fluorescence intensity of DOX. On the contrary, with O_2_ interference, the hypoxia distribution shrunk considerably, and DOX spread in the lower hypoxia area, indicating the O_2_ intervention of DHCNPs mitigated tumor hypoxia and enhanced the DOX accumulation in tumor (Figure 4c). Based on the tumor volume assessments, the coated NPs showed the highest tumor inhibition (Figure 4d), and mice treated with membrane‐coated NPs achieved 100% survival at the end of the experiment (Figure 4e). Overall, these NPs achieved enhanced targeted delivery of DOX and oxygen to tumor site without leading to adverse effects on healthy tissues. An important advantage of oxygen supply is that hypoxic milieu was significantly changed and the hypoxia‐induced chemoresistance was prevented by the suppression of HIF‐1α‐induced expression of the xenobiotic molecular pump—P‐glycoprotein (P‐gp), resulting in the enhanced DOX accumulation at the tumor site. The studies, both in culture and in vivo, showed that the synthesized biomimetic NPs killed tumor cells and inhibited the growth of solid tumors in animal models.^150^

CCMs were also used to coat mannose modified adjuvant PLGA NPs intended for anticancer vaccination, where PLGA NPs were first loaded with imiquimod (R837, receptor‐7 antagonist), and later coated with cancer cell‐derived membranes, of which surface proteins act as tumor‐specific antigens. The cell membrane‐coated NP vaccines were designed to limit metastasis and invasion into tissues. The coated particles showed greater uptake by dendritic cells (antigen presenting cells), leading the cells to mature and trigger antitumor immune responses.^151^ Vaccination of animal models with these NPs led to reduced cancer cell migration toward human mammary fibroblasts and reduced metastasis in vivo.^152^ The translocation of NPs to lymph nodes was confirmed by fluorescence imaging indicating directed immune stimulation. Accordingly, the use of these NPs can inhibit cancer cell‐stromal cell interactions and can prime the immune system as a cancer immunotherapy.

#### 3.1.4 MSC membrane‐coated NPs

MSCs have demonstrated preferential migration into tumor sites and selective homing to cancerous tissue in the body.^153^, ^154^ Therefore, the use of MSC membrane‐coated NPs may represent good candidate for delivery of chemotherapy due to this intrinsic tumor tropic property.^155^, ^156^ Cell migration is an active process requiring that the cell expend energy, this would be lost if the membranes are transferred to NPs, but the protein–protein interactions that facilitate adherence to abnormal vasculature and cancer cells could be transferred to NPs. In one study, umbilical cord MSC membrane‐coated PLGA NPs were used for tumor specific delivery of DOX.^153^, ^154^ NPs accumulated at the tumor site and were taken up by tumor cells, leading to tumor cell death. The use of MSC membranes significantly increased cellular uptake of NPs, tumor‐targeting, NP accumulation in the tumor and toxicity to tumor cells as compared to non‐coated NPs. In another study, bone marrow derived MSC membranes were used for the coating of paclitaxel‐loaded PLGA NPs,^157^ and the anticancer effect of these NPs was demonstrated in orthotopic glioma tumors in rats. NPs infiltrated gliomas and released their drug cargo into the tumors, demonstrating the targeted delivery of therapeutics to gliomas using MSCs membrane‐coated NPs.

#### 3.1.5 Platelet membrane‐coated NPs

Platelets have the innate ability to adhere to injured blood vessels and pathogens. Platelet membrane‐coated NPs can also evade the immune system; platelets have surface moieties that facilitate subendothelial adhesion, pathogen interaction and immune evasion.^141^ Such NPs can be used for targeted delivery of therapeutics agents. In a recent study, PLGA NPs were used for the delivery of verteporfin.^158^ The use of platelet membrane wrapping helps to develop targeted solar irradiation photodynamic therapy and helps to limit the effect of reactive oxygen species in the area defined by the NPs. The platelet membrane envelope provides NPs with active targeting capability. Because of its inherent property to adhere to collagen type IV, binding of platelet membrane wrapped docetaxel loaded PLGA NPs to collagen type IV was evaluated. Using fluorescent labeling, these NPs had significantly enhanced accumulation as compared to bare or RBC membrane‐coated NPs, indicating the platelet membrane‐type‐specific adhesion to collagen IV.^51^

#### 3.1.6 Hybrid cell membrane‐coated NPs

Hybrid cell membrane biomimetic NPs have several functionalities that can be enhanced in a single nanodrug delivery platform by the fusion of a combination of cell membranes. The strategy of fabricating hybrid cell membrane‐coated NPs improved the functionality in the treatment of different types of cancers, and such hybrid NPs accumulated at the target site at a greater degree.^159^, ^160^ The use of cancer cell‐macrophage hybrid membrane for coating of PLGA NPs was investigated for use in targeting breast cancer lung metastases.^161^ Overexpression of α4 and β1 integrins on RAW264.7 (macrophages) and 4T1 (breast cancer cells), and the presence of macrophage membranes led to enhanced targeting to lung metastasis.^161^ RBC‐platelet hybrid membrane coating of PLGA NPs enabled preservation of functional moieties characteristic of each cell source of membranes where they exhibited longer circulation time and demonstrated enhanced retention in human macrophage lines (THP‐1).^159^ Accordingly, their suitability for a variety of therapeutic and theranostic applications could be envisioned. Applications of different cell membrane‐coated PLGA NPs in cancer therapy are summarized in Table 2.

**TABLE 2:** Applications of cell membrane‐coated PLGA NPs in cancer therapy

No.	Cell membrane source	Drug/imaging agent	Key finding	Reference
1	RBCs	Antigenic peptide, and monophosphoryl lipid	Developed nanovaccine had better tumor prevention, growth, and metastasis. Dual antigen entrapment and stimuli‐responsive behavior.	^162^
		Arsenic trioxide	Enhanced circulation time and sustained release of arsenic trioxide and reduced toxicity. RBCs–PLGA arsenic trioxide NPs showed lower cytotoxicity than arsenic trioxide solution to normal 293t kidney cell lines and an antitumor effect against HL60 cells by CCK8 assay.	^163^
2	WBCs	Doxorubicin	Enhanced cellular uptake and cytotoxicity of doxorubicin loaded nano ghosts as compared to non‐coated NPs in breast cancer (MCF‐7) cell lines (in vitro).	^164^
3	Cancer cells	DiD fluorophore	In cancer immunotherapy, the facilitation of multiple antigens combined with immunological adjuvants was due to uptake of membrane bound tumor antigens for downstream immune activation. In anticancer therapy, cancer cell membrane coating provides homotypic binding mechanism.	^37^
		Indocyanine green	Dual imaging and photothermal therapy. The NPs successfully reduced the interruption of liver and kidney, the cell adhesion molecules on the surface of NPs possessed homologous targeting.	^149^
		Doxorubicin	Enhanced in vitro cellular endocytosis and antitumor effect toward HepG2 cells. Significantly enhanced in vivo antitumor effect and decreased systemic toxicity as compared to control (doxorubicin) owing to enhanced systemic circulation, enhanced doxorubicin accumulation at the tumor site and effective immune escape.	^165^
4	Stem cells	Paclitaxel	Mesenchymal stem cells loaded paclitaxel NPs showed effective killing of A549 lung cancer and MA 148 ovarian cancer cells in vitro. MSC membrane‐cloaked paclitaxel loaded NPs resided in the lungs (in vivo distribution studies).	^166^
		Doxorubicin hydrochloride	Better tumor homing and permeation properties. Better treated pulmonary metastases in C57BL6 mice. Mesenchymal stem cells loaded PLGA NPs reside in lungs for long time as compared to bare NPs (In vivo biodistribution studies).	^167^
5	Platelets	Docetaxel	Enhanced EPR effect slowed down the release of docetaxel from platelet membrane NPs and effectively suppressed the tumor growth in vitro. The platelet membrane coated with PLGA NPs showed enhanced circulation and tumor targeting. Significant suppression of tumor growth in A549 cell bearing nude mice (in vivo).	^168^
6	Combined macrophage and cancer cells	Metformin and siRNA targeting fibrinogen‐like protein 1 mRNA (siFGL1)	Effectively silenced the FGL1 gene. Promoted T‐cell‐mediated immune responses. Enhanced antitumor immunity.	^169^

Abbreviations: EPR, enhanced permeability and retention; MSC, mesenchymal stem cells; NPs, nanoparticles; PLGA, poly(lactic‐*co*‐glycolic acid); RBC, red blood cell; WBC, white blood cell.

### 3.2 Inflammation

Inflammation is a protective response, activating the immune system to protect the body from hazardous effects of pathogens, foreign bodies or disease,^170^ and the response may be acute or chronic.^171^ Immune activation that persists beyond the initial insult, which is due to a self‐antigen, or a result of nonspecific stimulation of immune cells leads to chronic inflammation that becomes the pathology. Such inflammatory conditions are characteristic of several disease conditions such as rheumatoid arthritis (RA),^172^ GI inflammatory disorders,^173^ cancer,^174^ pneumonia,^175^ and CVDs.^176^ Therefore, the development of novel tools that aid in the diagnosis and treatment of chronic inflammatory conditions are needed. NPs can be used to achieve this however, NPs can easily be detected and uptaken by phagocytic cells of the RES, which results in diminished effects.^177^ To overcome this problem, cell membrane‐coated NPs have been examined in models of chronic inflammatory diseases.^8^ From among the different types of cell membrane‐coated NPs, employing RBC, WBC, platelet, cancer cell or hybrid cell membrane coatings, and tumor exosome‐coated nanoparticles, each has been used in attempts control the inflammatory response.^178^

#### 3.2.1 Cancer‐associated inflammation

Inflammation is a hallmark of cancer, and tumors both elicit an immune response and attempt to control immune cell function for the malignancy to persist. WBCs are attracted to the tumor site and comprise a significant component of the tumor microenvironment. Together, the immune cells and the cancer cells then produce a myriad of chemokines and cytokines that both recruit and repel cells of the immune system. The complexity of the tumor microenvironment contributes to complex set chemical mediators including cytokines, cysteine proteases, reactive oxygen species that are variable with the stage of disease and type of cancer. Some of the secreted factors such as interferons, tumor necrotic factor (TNF)‐α, interleukins and membrane perforating agents, mediate cell death and lead to necrotic tissues within the tumor.^179^ Cancers are a site of chronic inflammation and the immune cell profile of the microenvironment with immune cells that are both pro‐ and anti‐inflammatory in nature.

**FIGURE 5:**
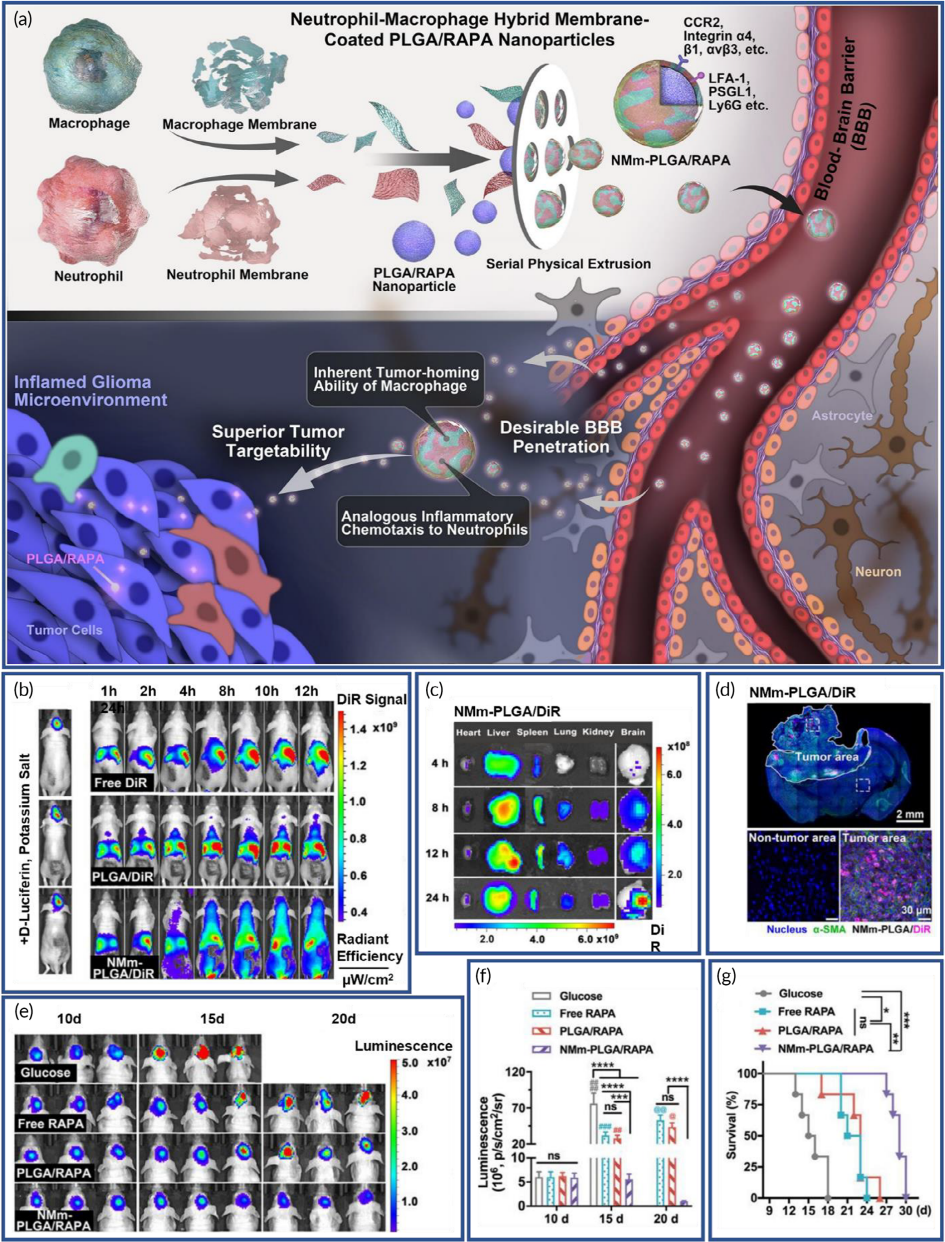
Dual membrane‐coated nanoparticles (NPs) with tumor‐microenvironment responsive ability for targeted delivery. (a) Schematics of a neutrophil‐macrophage hybrid membrane‐coated poly(lactic‐*co*‐glycolic acid)/rapamycin (PLGA/RAPA) nanoplatform. (b) Fluorescence tracking of DiR in C6‐Luc glioma‐bearing mice post injection. (c) Ex vivo fluorescence images of main organs and brain tissue after neutrophil and macrophage membranes (NMm) coated PLGA/DiR (NMm‐PLGA/DiR) treatment. (d) Fluorescence images of DiR and α‐SMA (microvasculature marker in glioma in green) in brain tumor at 24 h after NMm‐PLGA/DiR administration. (e) Bioluminescent images of tumor. (f) Quantification data demonstrating the glioma growth after treatment. (g) Survival curves. Reproduced with permission.^180^ Copyright 2022, Elsevier.

Rapamycin (RAPA)‐loaded PLGA NPs (NMm‐PLGA/RAPA) were coated with neutrophil and macrophage membranes (NMm) with BBB penetrating capability, and it combined the stimuli‐homing responsiveness of macrophage with the inflammatory chemotaxis of neutrophils (Figure 5a). Successful establishment of the mouse model was verified by a strong bioluminescent signal of glioma (Figure 5b). Following, real‐time in vivo biodistribution of each group was examined (Figure 5c). Ex vivo fluorescence imaging of the main organs reassured the excellent capability of NPs in brain targeting (Figure 5d). Using the immune staining results, it was also established that the NMm‐PLGA/DiR were explicitly targeted to the tumor site (Figure 5e), and the progress of tumor proliferation was clearly suppressed (Figures 5f,g). Besides, survival time of the mice was extended compared to the control group (Figure 5h).^180^

Granulocyte macrophage colony stimulating factor distinguishes monocytes into premature dendritic cells which then travel toward inflamed tissue and process tumor antigens to both recruit and activate T‐lymphocytes.^181^ Many of these processes require adherence molecules on the immune cells for them to be retained at the tumor location, therefore, cell membrane‐cloaked NPs may provide an opportunity for the targeted delivery of anticancer therapeutics.^182^ The vasculature of tumors is disrupted, irregular and leaky, thus cells and particles accumulate at the tumor site due to the effect known as EPR. Once cells and particles leak into the tumor microenvironment, proteins called adhesins, selectins and integrins allow them to be retained providing the opportunity to deliver their cargo; immune cell membranes are coated with these adherence molecules and coating particles with these membranes could confer the adherence properties. For these reasons, activated leukocyte membranes rich in inflammatory‐related receptors have been used for enhanced targeting of PLGA‐based nanovectors to inflammatory tumors.^183^ Drug loaded nanovectors that were coated with membranes from WBCs, thus are retained at the inflammation sites, improving their anti‐inflammatory effect by overcoming clearance by the liver to prolong circulation time, and increase the anticancer effects by increased accumulation in the tumor with subsequent release of the drug. When neutrophil membranes were used for the coating of PLGA NPs, the desired bioactive functions were preserved.^146^ Compared to uncoated NPs, coated NPs showed enhanced binding to circulating tumor cells and improved the localization of drug at the sites of inflammation associated with mouse mammary carcinomas. Moreover, membrane‐coated PLGA NPs containing carfilzomib (a proteasome inhibitor) specifically depleted circulating cancer cells in blood and as a result, inhibited early metastasis and effectively stopped the growth of previously formed metastasis in mouse models. Platelet membranes were also investigated for coating PLGA NPs, and these unilamellar membrane‐coated particles displayed immunomodulatory' antigens on their surface including glycoprotein IV, V, VI, and XI, CD59, CD47, α2, β1, α5, β3, and GPIbα. These platelet membrane‐coated NPs mimicked the adhesion properties of platelet and bound to sites of vascular damage and reduced macrophage uptake.^51^ In another study, hybrid RBC‐platelet membrane‐coated PLGA NPs were found to also improve NP properties by targeting inflammation and providing extended blood circulation while offering lower production costs.^159^

#### 3.2.2 Arthritis

RA is a chronic systemic autoimmune synovitis characterized by joint pain, cartilage destruction and progressive loss of function in multiple joints.^184^ RA has a multifactorial etiology with implicated genetic and environmental factors that lead to activation of endothelial cells. This, in turn, causes stimulation of NK cells, neovascularization, accumulation of fibroblast‐like macrophages and mast cells, and a characteristic hyperplastic synovial layer.^185^, ^186^

**FIGURE 6:**
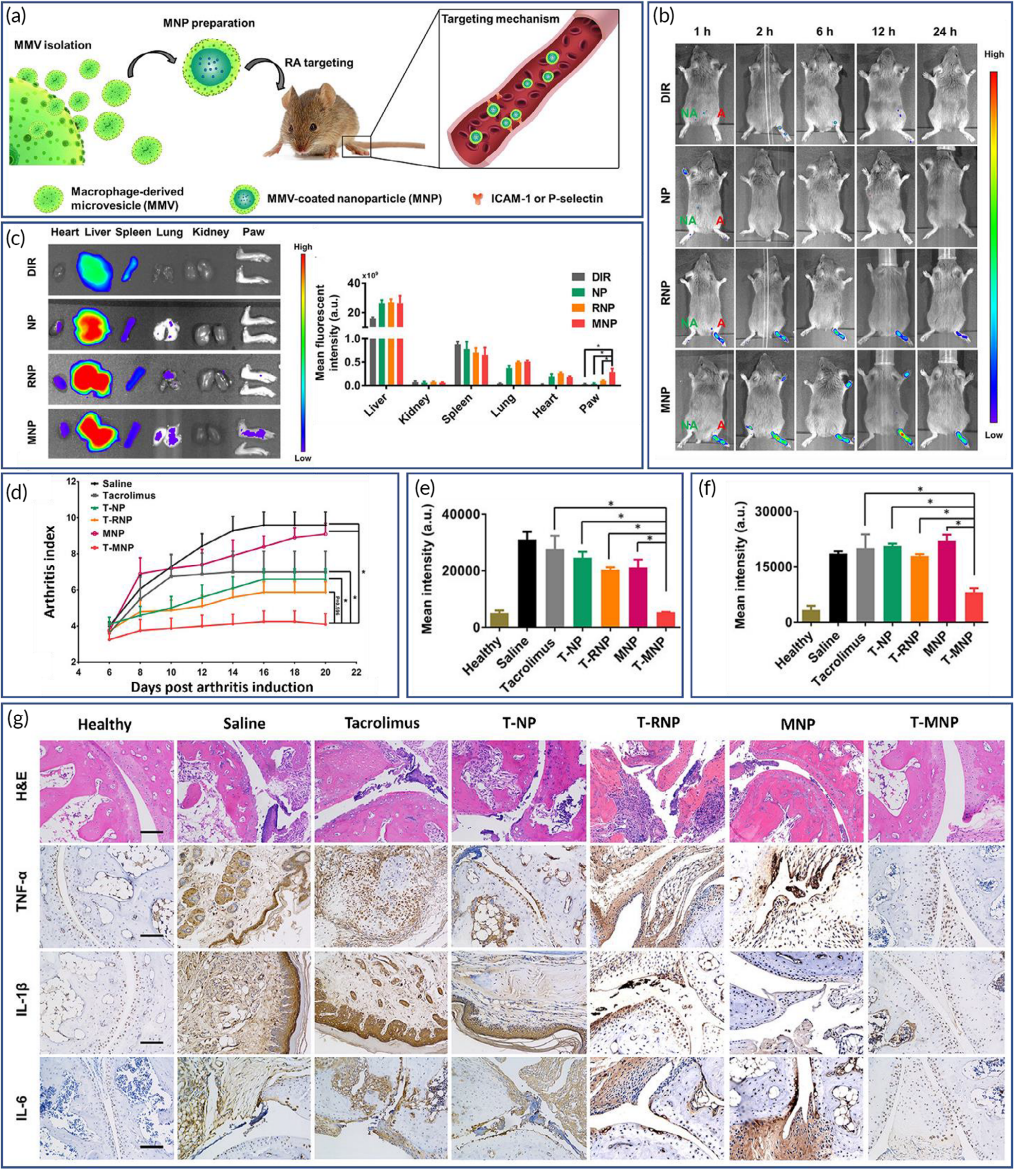
Targeted delivery of therapeutics to sites of rheumatoid arthritis (RA). (a) Schematic representation of macrophage‐derived microvesicle‐coated poly(lactic‐*co*‐glycolic acid) (PLGA) nanoparticle (MNP) targeting sites of rheumatoid arthritis (RA). (b) Targeting effect of MNP to sites of RA. Accumulation of different nanoparticles (NPs) in arthritic paws or nonarthritic paws. (c) Distribution of MNP in various organs compared with free DIR (dye), bare NP, and RNP (red blood cell membrane‐coated nanoparticle). (d) Fluorescence intensity of MNP in different organs. (d) Arthritis index in different groups over 14 days of treatment. (e, f) Mean intensity of TNF‐α and IL‐1β staining in different groups. (g) Immunohistochemical image of synovium in different groups; scale bar, 100 μm. Reproduced with permission.^190^ Copyright 2019, American Chemical Society.

Inflammation is associated with activation of kB ligand (RANKL), pro‐inflammatory cytokines (TNF‐α), interleukins (IL‐1 IL‐6), prostaglandins, TNFs, and reactive oxygen intermediates.^187^ Nonsteroidal anti‐inflammatory drugs (NSAIDs) comprise the standard of care, but when used at high doses for long durations, these and other anti‐rheumatic drugs have serious side effects. Treating chronic diseases will require targeted delivery to spare normal tissues.^187^, ^188^ In one study, PLGA NPs containing the drug FK506 (a model drug for RA' therapy) were coated with platelet‐membranes with the intent of platelet membrane proteins (GPVI) interacting with overexpressed CD44 and collagen IV in synovial tissues to facilitate local accumulation and drug release.^79^ Coated NPs had improved stability, prolonged circulation, and a near twofold greater retention at sites of inflammation then did uncoated MPs. Other cell membranes including those from neutrophils^189^ and macrophage‐derived microvesicles (MMVs)^190^ showed targeted delivery to inflammed joints and neutralization of pro‐inflammatory cytokines in RA. An MMV‐coated NP (MNP) was developed for targeting RA (Figure 6a). While MNP was effectively targeting, non‐coated NPs were unable to reach paws of mice (Figures 6b,c). Therapeutic effect of the drug loaded MNP (T‐MNP) was validated by intravenous injection of NPs (Figure 6d), through the histological examination of pro‐inflammatory cytokine expressions (Figures 6e,f) and immune histochemical analysis (Figure 6g).

#### 3.2.3 GI inflammatory conditions

The inflammatory response in the GI tract is used to eliminate pathogens and toxins and is usually self‐limiting.^191^ However, loss of immune control can lead to IBD, and loss of homeostasis in this chronic condition leads to progressive tissue damage with leakage of gut contents into the abdominal cavity which increases inflammation.^192^ To stop this cycle and restore immunological homeostasis, a number of probiotics, corticosteroids, NSAIDS and other immunosuppressive agents have been tried, but these typically fail, and new treatment strategies are needed for the management of IBD and other inflammatory diseases of the GI tract. Biomimetic nanocarriers naturally functionalized with cellular adhesion molecules from cell membranes have been investigated for a number of models of chronic inflammatory diseases of the GI tract.^58^

**FIGURE 7:**
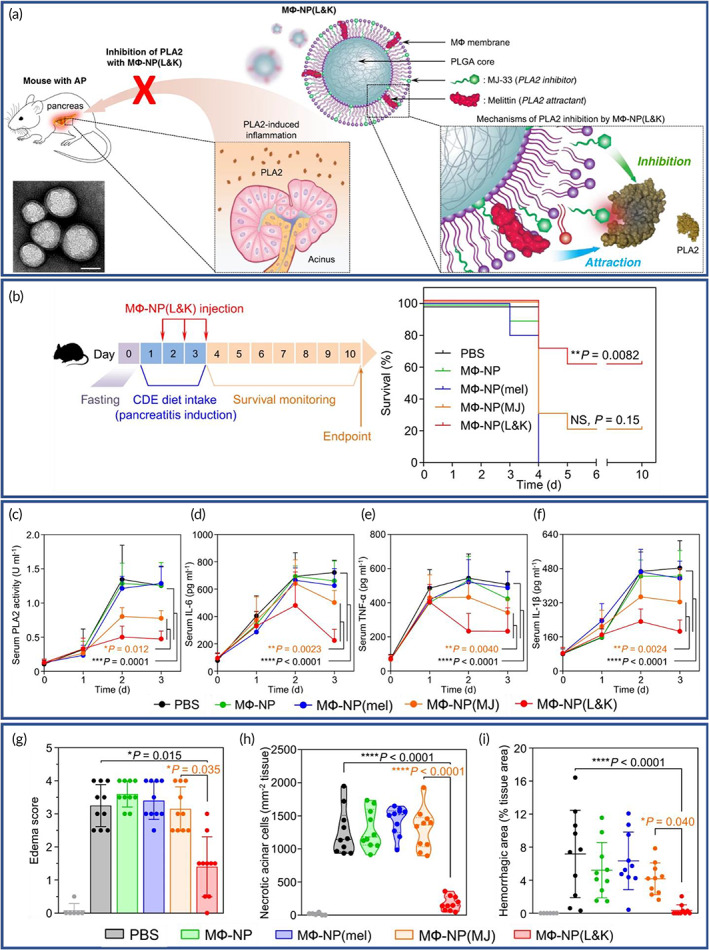
Macrophage membrane‐coated nanoparticles for the treatment of acute pancreatitis. (a) Schematic representation of phospholipase A2 (PLA2) inhibition during acute pancreatitis development by macrophage membrane‐coated nanoparticles with a built‐in “lure and kill” mechanism referred to as MΦ‐NP(L&K). Inset shows the TEM image of MΦ‐NP(L&K). Scale bar, 100 nm. (b) The study protocol of lethal pancreatitis induction and treatment with MΦ‐NP(L&K) and survival rates of mice over 10 days after initial diet intake. (c–f) PLA2 activity profiles in serum of acute pancreatitis mice treated with different formulations. Concentration levels of inflammatory cytokines, including IL‐6, TNF‐α, and IL‐1β. (g–j) Edema score, necrotic cell levels, hemorrhagic area, and CD45^+^ cell levels in acute pancreatitis mice treated with different formulations. Reproduced with permission.^195^ Copyright 2021, Nature.

In one study, gastric epithelial cell line‐derived membranes were used for coating of PLGA NPs with antimicrobial agents to treat *Helicobacter pylori* infections.^193^ These NPs were loaded with clarithromycin and exhibited superior therapeutic efficacy and reduced the GI inflammation when they were compared to non‐coated NPs or free drug. In another study, neutrophil membranes were used to coat PLGA NPs containing celastrol and evaluated for the treatment of acute pancreatitis.^194^ These NPs specifically accumulated in the pancreas and selectively distributed in the pancreatic tissues better than noncoated NPs. Pancreatic acinar cells produce phospholipase A2 (PLA2), which is known as trigger of acute pancreatitis. Therefore, macrophage membrane‐coated PLGA NPs doped with melittin (a short peptide that can trap PLA2 to attack the membrane) and MJ‐33 (PLA2‐specific inhibitor) were developed to direct therapy to PLA2 expressing cells in the treatment of acute pancreatitis (Figure 7a,b).^195^ The MJ‐33 and melittin work as PLA2 obstructor and PLA2 attractant respectively. These NPs could counteract PLA2 activity in a dose‐dependent manner, and depress PLA2‐activated inflammatory responses (Figure 7c–f). Compared to control groups, notable reduction in edema score, counts of necrotic acinar cells and hemorrhagic area in the pancreatic tissue were observed with NPs (Figures 7g–i).

### 3.3 Cardiovascular diseases

CVD has the highest morbidity and mortality worldwide,^196^ and disorders such as cardiac and cerebral ischemia^197^ and atherosclerosis^198^ are largely associated with vascular inflammation.^199^ The foremost causes of disability and death in the developed world have long been CVDs and their sequelae,^200^ and prevention has been the focus of many treatment strategies. Although the cholesterol‐lowering drugs have been used successfully for the past 25 years, novel treatment protocols focusing on vessel wall inflammation have also been examined,^201^, ^202^, ^203^ and targeted therapy can be employed.^8^, ^204^, ^205^, ^206^

#### 3.3.1 Atherosclerosis

Atherosclerosis is a gradual inflammatory disease that has a lipid and fibrous artery wall build‐up, and is a significant cause of mortality and morbidity.^207^ Currently, atherosclerosis is treated with both medicines and surgical intervention. Treatment of early‐stage atherosclerosis is restricted to general oral medications.^208^ Even though procedures such as stenting are effective for treating progressive atherosclerosis, these procedures are associated with side effects, such as restenosis and thrombosis.^209^, ^210^ On the other hand, nanotechnology enables the specific targeted delivery of therapeutic compounds, has the potential to increase efficacy and safety,^211^, ^212^, ^213^ and NPs with a biomimetic natural surface can avoid the RES and can be used for specific targeting.^214^, ^215^

Atherosclerosis is a disease driven largely by macrophages. When these cells accumulate in the vessel wall, they secrete cytokines that recruit additional immune cells and metalloproteinases leading to thrombosis.^216^ Conventional therapies have been ineffective due to poor delivery and the need for long‐term administration at high doses that are associated with serious side effects.^217^ Nanoparticulate drug delivery systems such as nanocapsules, nanoemulsions, lipid NPs, polymeric NPs, hybrid NPs and metallic or inorganic nanocarriers have been investigated for targeted drug delivery for vascular inflammation, but none, to date, have demonstrated efficient targeting.^218^ Biomimetic drug nanocarriers may offer hope for effective treatment of cardiovascular inflammation.^219^ In one study, macrophage membrane‐coated PLGA NPs were investigated and found to recognize the VCAM‐1 receptor in cultures of human vascular endothelial cells and to have accumulate in atherosclerotic lesions in the Apo E^−/−^mouse model.^220^

**FIGURE 8:**
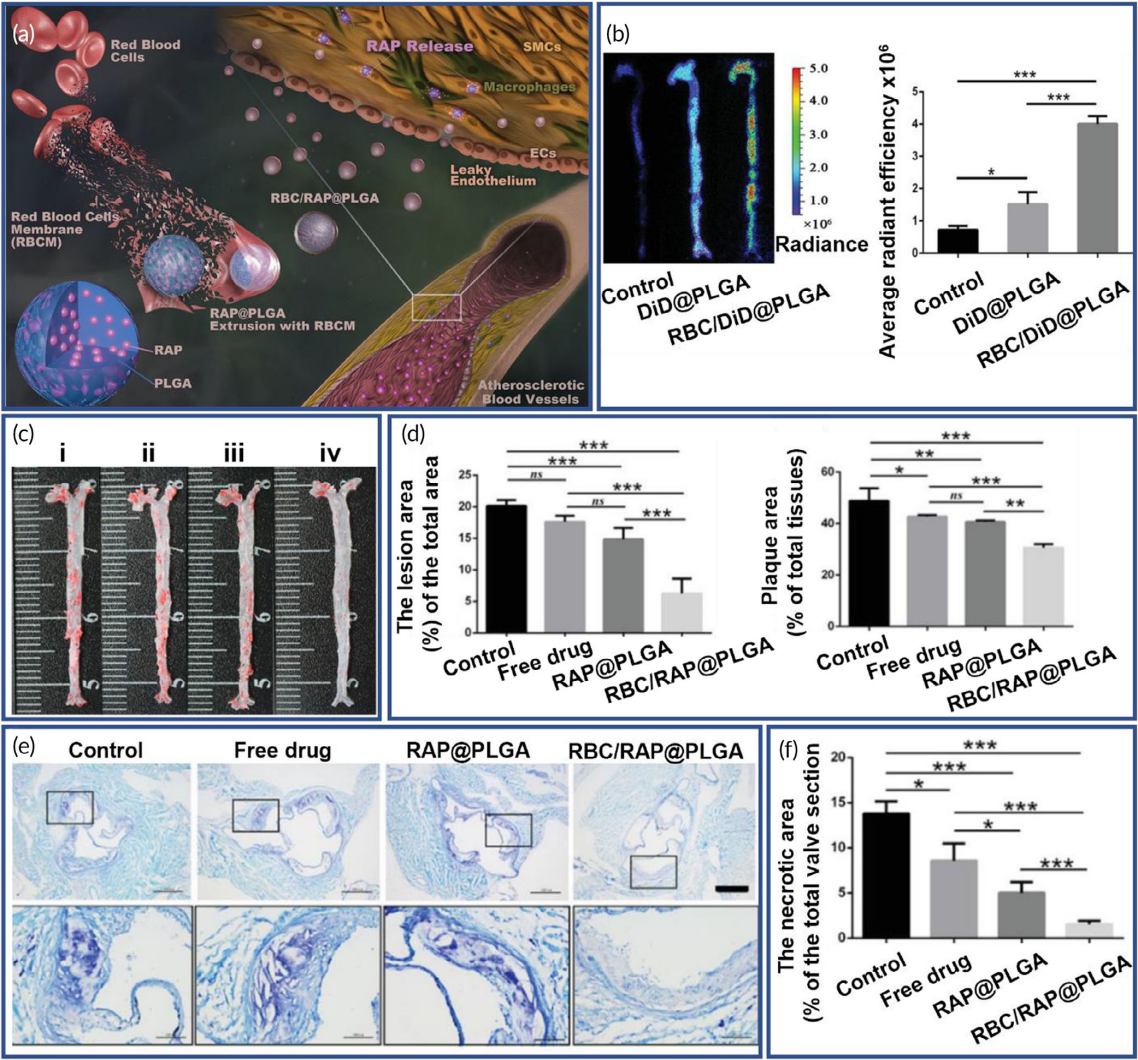
RBC membrane‐coated nanoparticles (NPs) for the treatment of atherosclerosis. (a) Schematic representation of red blood cell membrane‐coated rapamycin loaded poly(lactic‐*co*‐glycolic acid) (PLGA) NPs (RBC/RAP@PLGA) for atherosclerosis management. (b) The ex vivo fluorescence images and the quantitative data of the aorta of mice which was fed with a high‐fat diet for 10 weeks. (c) Stained images of aorta and aortic roots sections. (i) Control, (ii) free drug, (iii) RAP@PLGA, and (iv) RBC/RAP@PLGA. (d) Quantitative data of the atherosclerotic plaque area. (e) Images of the necrotic areas stained by Toluidine blue (scale bar = 500 μm). (f) Quantitative data of the necrotic areas in the aortic root sections. Reproduced with permission.^217^ Copyright 2019, John Wiley & Sons.

RAPA has been investigated as anti‐atherosclerotic drug but exhibits low concentrations in atherosclerotic lesions; to achieve efficacy, high doses were used which led to sever systemic side effects. For the management of atherosclerosis, PLGA NPs were first loaded with RAP, later coated with RBC membrane, and RBC membrane coating of RAPA loaded PLGA NPs were evaluated for targeted drug delivery (Figure 8a).^217^ Atheroscleros is was induced in mice, and NPs were injected through tail vein. While RBC/DiD@PLGA accumulated in atherosclerotic plaque areas, the DiD@PLGA group exhibited considerably lower signals in atherosclerotic plaque compared to the RBC/DiD@PLGA group (Figure 8b). The antiatherosclerosis potential of free RAP, RAP@PLGA, and RBC/RAP@PLGA in mice was tested by collecting and staining the aortas after treatment for 1 month. RBC/RAP@PLGA treatment resulted in substantially higher therapeutic efficacy than RAP and RAP@PLGA (Figure 8c,d). Immunohistochemistry staining was used to examine the necrotic areas in the aortic roots, where the control group showed large necrotic areas with substantial cholesterol crystals, implying advanced lesions. Application of RBC/RAP@PLGA decreased the necrotic areas significantly, further confirming the efficacy of the treatment (Figures 8e,f). Overall, these biomimetic RBC membrane‐coated PLGA NPs significantly diminished the progression of atherosclerosis.

Platelets have an inherent affinity for atherosclerotic plaques, and RAPA loaded PLGA NPs coated with platelet membranes demonstrated targeted drug delivery and significantly reduced the progression of atherosclerosis in mouse models.^77^ Lumbrokinase is another drug used to treat thrombotic diseases, but nonspecific delivery is associated with hemorrhagic (bleeding) complications.^221^ Therefore, platelet membrane‐coated PLGA NPs loaded with lumbrokinase were developed to risk and overcome the short half‐life of the drug. These NPs demonstrated selective adherence to thrombotic plaques, had thrombolytic activity, and reduced adverse effects compared to free drug.

In another study, macrophage membrane coating was applied to the surface of RAPA‐loaded PLGA NPs.^222^ Improved biocompatibility of the NPs in comparison with naked NPs, was demonstrated in blood compatibility analysis in vitro. Coated NPs efficiently reduced macrophage phagocytosis and in vitro targeting of activated endothelial cells. Furthermore, they successfully targeted and resided in atherosclerotic lesions in mice. They were found to dramatically slow the course of atherosclerosis after a 4‐week treatment regimen. Furthermore, long‐term administration of the coated NPs revealed acceptable safety results. To achieve explosive drug release from PLGA NPs, macrophage‐derived membrane was used to coat responsive ROS‐cores comprised of PLGA.^223^ Coating not only made escape of NPs from RES easier, but it also helped achieve targeted delivery to atherosclerotic lesions where loaded drugs can be released fast because of the oxidative environment.

Hybrid NPs composed of lipid/apolipoprotein (APoE) shell and PLGA core were recently developed.^224^ These new HDL‐like NPs exhibited preferential uptake by macrophages and an efflux capacity of good cholesterol. In an ApoE knockout atherosclerotic mouse model, NPs have accumulated in atherosclerotic plaques, and they were localized along with plaque macrophages. Use of platelet membranes to coat PLGA NPs loaded with paclitaxel was investigated in an experimental rat model of coronary artery restenosis.^50^, ^225^ However, precise distribution of various platelet membrane glycoproteins on the platelet membrane cannot be controlled due to the limited number and partial functionalization of proteins present on platelet coated NPs. As a result, these NPs were not well suited for targeting because of short cycling times, but their efficiency was seven to eight times higher than that of noncoated NPs. Collectively, these studies show that membrane‐coated NPs can effectively and safely slow the development of atherosclerosis. These advances in delivering anticoagulant and antiplatelet drugs have tremendous promise for treating CVD.

#### 3.3.2 Ischemia

Stroke is the third leading cause of mortality in the United States, causing almost 150,000 deaths per year.^226^ Ischemic stroke is the most frequent type of stroke and happens when blood supply to the brain is interrupted due to thrombosis.^227^ The presence of biological barrier (BBB) is the major hurdle in clinical management of stroke. In later stages of stroke, the integrity of BBB is partially disrupted, but it remains largely intact within the therapeutic window and may not allow the transfer of pharmacologically required drugs for effective treatment. Nanotechnology can enhance the brain penetrability, but existing approaches have been shown to be inadequate.^228^ Therefore, biomimetic NPs may provide means to cross BBB, and increase the accumulation of drugs at the sites of disease, as a result they can be used as innovative diagnostic systems for early stage stroke diagnosis, for example to detect several biomarkers (ROS and neurotransmitters).^78^, ^229^, ^230^

**FIGURE 9:**
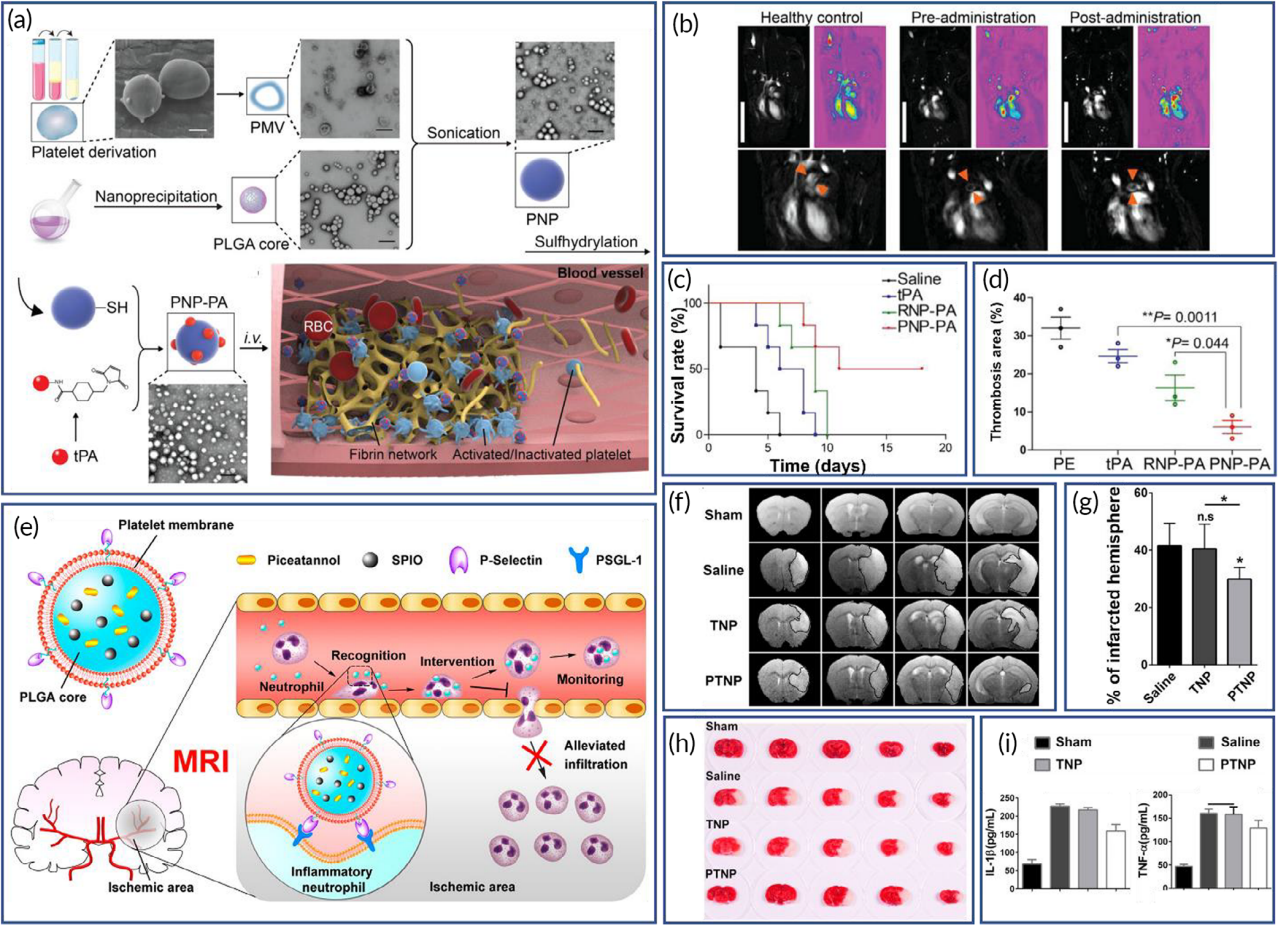
Platelet membrane‐camouflaged nanoparticles (NPs) for targeted delivery of the thrombolytic drug local thrombus sites (a–d), and for monitoring and treatment of acute ischemic stroke (e–i). (a) Schematics of the synthesis of platelet membrane‐cloaked polymeric NPs (PNPs) conjugated with rt‐PA (tPA) on the surface referred to as PNP‐PA. (b) T1 magnetic resonance imaging (MRI) image of mice before and after NPs treatment. Scale bar: 2 cm. (c) Survival rate of pulmonary embolism (PE) mice treated with the different drug formulations. (d) Quantification of the thrombus area in part (c). Reproduced with permission.^232^ Copyright 2019, John Wiley & Sons. (e) Schematic representation of platelet‐mimetic nanoparticles containing poly(lactic‐*co*‐glycolic acid) (PLGA) core co‐loaded with piceatannol and superparamagnetic iron oxide (SPIO). Post intravenous injection, platelet‐mimetic nanoparticles (PTNPs) could selectively distinguish inflammatory neutrophils and they were internalized into adherent neutrophils where the loaded piceatannol was released, thus supporting the detachment of neutrophils from endothelial cells into circulation, resulting in reduced neutrophil infiltration. Due to SPIO, MRI could image inflammatory neutrophils. (f) T2‐weighted images of ischemic brains, where the black curve refers to the infarct region. (g) Quantification of the infarction volumes. (h) Stained images of brain slices. The white region indicates the infarct region. (i) Expression degrees of IL‐1β and TNF‐α in the brains of mice. Reproduced with permission.^236^ Copyright 2019, American Chemical Society.

Ischemia of the heart is another leading cause of death worldwide, and for the diagnosis of myocardial ischemia reperfusion injury, platelet membrane‐coated PLGA NPs have been developed.^231^ Coated NPs showed enhanced in vitro and in vivo performance as compared to noncoated NPs. Furthermore, localization to the target area was achieved as indicated by echo signal intensity, which was significantly higher in the risk area as compared to remote areas of the myocardium in rat models. An immunofluorescence assay and ex vivo fluorescence imaging was used to validate these findings. Fluorescent signals indicated consistent accumulation of the NPs at the target site and their slow degeneration. In another study, recombinant tissue plasminogen activator rtPA (used for lysis of established thrombi) PLGA NPs enclosed in platelet membranes were developed for the targeted delivery to thrombus sites (Figure 9a).^232^ The usefulness of these NPs in reverse thromboembolism was demonstrated using MRI experiments (Figure 9b), and it was concluded that NP treatment improved the median survival time (Figure 9c) and dissolved the thrombi effectively (Figure 9d).

In the pulmonary embolism mouse model, this NP formulation specifically targeted the pulmonary thrombus and produced local clot degradation, leading to greater survival rate of mice and decreased bleeding risk as compared to the use of free rtPA. These NP formulations were also studied in mouse model of mesenteric arterial thrombosis and ischemia. In mice with mesenteric thrombosis, NPs demonstrated enhanced thrombolysis and improved the survival rate of mice when compared to free rtPA. To accomplish improved endothelial cell barrier penetration and increased retaining time in ischemic tissues, cell membranes obtained from human adipose‐derived stem cells that were transduced with mRNA vector to overexpress CXCR4 and used for the coating of vascular endothelial growth factor loaded PLGA NPs.^233^, ^234^, ^235^ Therapeutic outcome was dramatically improved, resulting in a 17% lower chance of limb loss compared to the untreated group (83%) in mice with hind limb ischemia. Platelet membranes were also used for the coating of SPIO NPs (SPIONs) and piceatannol (PTNPs) loaded PLGA NPs (Figure 9e). Having P‐selectin (platelet) and PSGL‐1 in the coating enabled the adherence of NPs to neutrophils. Released piceatannol led to alleviated infiltrations of neutrophils in the brain ischemic regions. In comparison with noncoated NPs, PTNPs significantly decreased the infarction volume in mice.^236^ Lower neutrophil infiltration is closely related to therapeutic effects in acute ischemic stroke. The mice treated with PTNPs showed considerably reduced neutrophil infiltration compared to control groups, and they also showed higher neurological scores and reduced infarct volumes (Figure 9f–h). After the PTNP treatment, the levels of inflammatory factors (TNF‐α and IL‐1β) in infarct half‐brains were greatly diminished (Figure 9i), indicating the alleviation of inflammation and effectiveness of the treatment.

### 3.4 Infectious diseases

Infectious diseases are accountable for majority of the hospitalization and are a prime cause of mortality and morbidity around the globe.^237^ The effective treatment of infectious disease remains a challenge due to drug resistance and the absence of specificity and effective drug availability. Antibiotic resistance can develop when levels of drug are insufficient to completely control bacterial growth and resistance remains a major challenge in antibacterial and antiviral therapy.^238^ Overuse of antibiotics used for the treatment of infection contributed to the development of drug‐resistant strains^239^, ^240^ for which nanomedicine and targeted antibody delivery options are being evaluated as alternative effective antibacterial approaches.^241^ Therefore, drug delivery systems that confine delivery to sites of infection, diminishing development of resistance, and reduce off‐target effects have been proposed and developed.^242^ Biomimetic nanocarriers have been developed which are derived from different mammalian cells, bacteria and viruses and vary in their construction and physicochemical properties.^243^

#### 3.4.1 Bacterial infections

**FIGURE 10:**
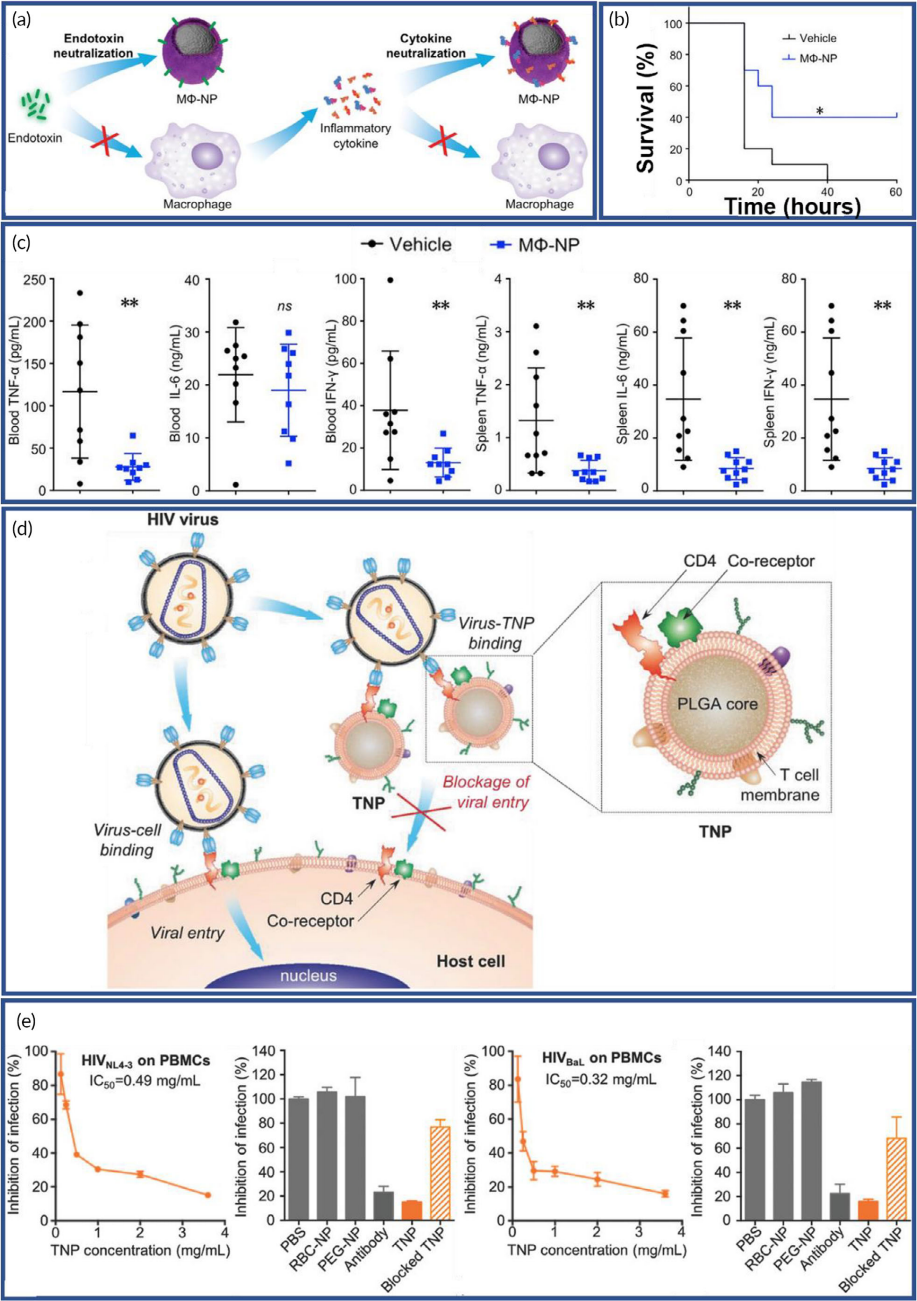
Macrophage membrane‐coated nanoparticles (NPs) for sepsis control (a–c), and T‐cell membrane‐coated NPs for mitigating HIV infectivity (d, e). (a) Schematics of macrophage membrane‐coated NPs (MΦ‐NPs) to neutralize endotoxins and pro‐inflammatory cytokines as a two‐step process for sepsis management. (b) Survival time of mice with bacteremia after treatment with MΦ‐NPs. (c) Quantification of pro‐inflammatory cytokines. Reproduced with permission.^73^ Copyright 2017, Proceedings of the National Academy of Sciences of the United States of America. (d) Schematics of T‐cell‐membrane‐coated NPs (TNPs) designed for mitigating HIV infectivity. TNPs were constructed by wrapping polymeric cores with natural CD4^+^ T‐cell membranes, which contain key antigens including CD4 receptor and CCR5 or CXCR4 coreceptors for viral targeting. By replicating the surface antigen profile of source T cells, TNPs can act as baits to bind with T cell targeted viruses and block viral entry into and infection of the target cells. (e) Effectiveness of TNPs in the inhibition of HIV infection. Reproduced with permission.^272^ Copyright 2018, John Wiley & Sons.

Sepsis is a systemic bacterial infection that can lead to multiple organ failure or dysfunction due to an uncontrolled inflammatory response, resulting in high mortality and morbidity.^244^ Effective treatment of sepsis is challenging and care is often dependent on supportive measures.^245^ Recently, macrophage mouse membrane‐coated PLGA NPs were developed and investigated for the treatment of sepsis (Figure 10a).^73^ These NPs aim to seize pro‐inflammatory cytokines and prevent their capability to enhance the spreading of sepsis. In a mouse model of *Escherichia coli* bacteremia, these NPs were found to achieve promising detoxification of endotoxins, inhibit bacterial spreading, leading to a significant increase in the survival of mice (Figure 10b) and considerable reduction of pro‐inflammatory cytokines (Figure 10c). Thus, these NPs have the potential to be used in the of sepsis management.

Group A Streptococcus is a gram‐positive bacterial pathogen responsible for a large number of diseases ranging from minor to severe infections. RBC membrane‐coated PLGA NPs have recently been investigated to protect macrophages, neutrophils and keratinocytes from the cytotoxic steptolycin O produced by group A streptococcus bacteria. Local administration of these NPs in a murine model showed decreased bacterial colony forming units and reduced lesion size, showed targeted therapy in severe streptococcal infections.^67^ Another gram‐positive bacterium responsible for a large number of infections is *Staphlococcus aureus* ^246^ and methicillin resistant strains (MRSA) are a significant concern.^247^ *S. aureus* secretes α‐toxin, a membrane destructive pore‐making toxin, which has various cellular targets such as leukocytes, epithelium, platelets, and endothelium.^248^

*Helicobacter pylori* infects over half of the global population,^249^ and is a major cause of gastritis, digestive ulcers, and gastric cancer. Currently, a triple therapy comprised of clarithromycin, proton pump inhibitor and an antibiotic either metronidazole or amoxicillin is the suggested treatment protocol.^250^ Drug resistance mutations in *H. pylori* have been found which make it resistant to these antibiotics,^251^ new and effective treatments are being sought.^252^ These include gastric epithelial cell membrane‐coated clarithromycin containing PLGA NPs, which were found to exhibit superior binding to *H. pylori* and accumulation at the target site as compared to noncoated NPs.^193^ Moreover, coated NPs showed prolonged drug release, high drug loading and greater bactericidal effect. Consequently, these NPs effectively decreased bacterial burden in a mouse model, and they are considered as safe and effective strategy that can be used for targeted delivery of the antibiotics to *H. pylori* infection sites.

Human platelet membrane‐coated PLGA NPs have been used to preserve host cell defense functions against *S. aureus* toxin; these coated NPs showed enhanced killing of drug‐resistant *S. aureus* via improved cytoprotection and toxin neutralization.^248^ Tedizolid phosphate (TR‐704) is an effective antibiotic approved for use in the treatment of acute skin infections caused by gram positive bacteria such as MRSA and methicillin‐sensitive *S. aureus* ^253^; and several drug delivery systems have been explored.^254^ Using an RBC membrane coating, PLGA NPs loaded with TR‐704 avoided uptake by macrophages which led to prolonged drug circulation.^255^

*Pseudomonas aeruginosa* is a gram‐negative bacterium and is responsible for lung infections (pneumonia), especially in cystic fibrosis and immunocompromised patients.^256^ Vaccines offer the greatest hope for prevention and are being actively investigated.^257^ One strategy for vaccination against Pseudomonas uses macrophage‐coated PLGA NPs to elicit potent immunity.^258^ This vaccine was established to be inherently multi‐antigenic, displaying wide range of *P. aeruginosa* antigens and safe for in vivo administration. The designed nanovaccine holds the natural ability of macrophages to neutralize virulence factors secreted by bacteria and in clearing of pathogens. Moreover, its administration in pneumonia induced in mice led the development of strong humoral immunity, which remarkably weakened the severity of infection caused by *P. aeruginosa*.

#### 3.4.2 Viral infections

Viral infections are responsible for universal fatality on a large scale.^259^ SAR‐CoV‐2 is a highly infectious human pathogen that causes a disease called corona virus disease 2019 (COVID‐19) which is a severe respiratory syndrome. This pandemic has led to unimaginable morbidity and mortality worldwide with half a million deaths in the United States in 2021.^260^, ^261^ There have been substantial efforts to find safe and effective vaccines, and by July 2021, 18 vaccines have been approved for emergency use.^262^ Cytokine storm syndrome (CSS) with tremendous inflammation leading to organ failure is a primary cause of death by SAR‐CoV‐2.^263^ Macrophages are a key cell type accountable for initiation and sustaining CSS in COVID‐19. Macrophages at the sites of viral infection are activated to produce chemokines and cytokines, resulting in recruitment of large number of immune cells fueling inflammation and CSS often leading to thrombosis.^264^ To improve targeting of antiviral agents, macrophage membrane‐coated lopinavir (LPV) loaded PLGA NPs have been investigated.^265^ The coating competitively absorbs many inflammatory materials, delivers the drug to sites of infection and prevents the development of CSS. Moreover, these NPs have shown improved antiviral efficacy compared to uncoated NPs. Additionally, the survival rate of studied mice (coronavirus infection model) has greatly increased after treatment with these NPs. Collectively, these NPs showed promising targeted antiviral and anti‐inflammatory effects with good outcomes in mouse models of COVID‐19. The infectivity of SARS‐CoV‐2 mostly occurs due to binding of the virus to known or unknown protein receptors on the target cells. Inspired by this, Zhang et al. developed cellular nanosponges as a medical countermeasure to the coronavirus. These nanosponges are composed of PLGA NPs surrounded by cell membrane derived from human cells (human lung epithelial type II or human macrophages) which are natural targets for coronavirus. These nanosponges display both known and unknown protein receptors required by coronavirus for cellular entry. Both cellular nanosponges neutralized SARS‐CoV‐2 in concentration dependent manner. The macrophage based nanosponges may have offer great potential as a therapy option because of their ability to reduce the viral load in the body, and address the severe and sudden inflammatory response at later stages of COVID‐19, due to inherent immunological functions of macrophages.^266^

Due to the high interest in mRNA delivery since the advent of mRNA based COVID‐19 vaccines, cell membrane coating technology was utilized for the cytosolic delivery of mRNA. Genetically engineered cell membrane expressing influenza A virus protein (hemagglutinin) was used to coat mRNA loaded PLGA NPs. The resulting biomimetic NPs mimic the ability of some viruses to achieve endosomal escape, which is the key obstacle in mRNA nanodelivery. The virus‐mimicking NPs successfully delivered the mRNA into the cytosolic compartment of target cells in vitro, resulting in expression of the encoded protein. Moreover, the expression levels of the protein were significantly enhanced in both local and systemic delivery of these NPs in vivo.^267^

The human immunodeficiency virus (HIV) is the cause of acquired immunodeficiency syndrome and treatment has only recently become effective.^268^ Although antiviral therapy is efficient in managing plasma virus at imperceptible levels, this therapy must be taken for lifetime, causing severe adverse effects and drug resistance.^269^ Moreover, discontinuation of this therapy results in the relapse of HIV infection within days or weeks.^270^ Approaches to eliminate the virus will require innovations that address both active replicating virus and the viral DNA that has integrated into the genomes of host cells and remains latent. The advantages of nanotechnology including increased half‐life in the circulation, improved patient tolerance and augmented efficacy of the anti‐viral drugs may benefit patient care.^271^ Cell membrane‐coated NPs have been investigated as another tool in the control of viral infections (Figure 10d).^272^ CD4^+^T lymphocyte cell membrane‐coated PLGA NPs bind glycoprotein gp120 on the virion itself or on the surface of infected cells. When these NPs bind HIV virions, they prevent gp120 from interacting with cells, and in culture, this reduced apoptosis of CD4^+^T cells. Infectivity was reduced for two strains of HIV (Figure 10e). These studies suggest that when combined with combination antiviral therapies, the long circulation times of cell membrane‐coated NPs may provide protection from newly released virus particles. If this approach was combined with drugs that reactivate the integrated proviruses from latently infected cells, opportunities for eliminating the virus may be possible.

**TABLE 3:** Applications of different cell membrane‐coated PLGA NPs

No.	Coating	Core Material	Experimental model	Outcome	Applications	Reference
1	RBC membrane	PLGA NPs	EL4 cells and male C57BL/6 mice bearing EL4 cells	Enhanced biocompatibility and immune eviction Enhanced efficacy in terms of tumor growth inhibition as compared to the use of free doxorubicin in a murine lymphoma model	Cancer	^143^
2	Macrophage membrane	PLGA NPs	Cellular model of SARS‐CoV‐2 pseudovirus infection and coronavirus infectious mouse model	Coating competitively absorbed many inflammatory materials, delivered drug into infection site Improved antiviral efficacy compared to uncoated NPs	COVID‐19	^265^
3	Platelet membrane	PLGA NPs	The human rheumatoid arthritis synovial cell line (MH7A) and CIA animal model	Targeted delivery to rheumatoid arthritis due to interaction of platelet membrane proteins (GPVI) with overexpressed CD44 and collagen IV in synovial tissues Improved NP stability, long circulation profile and provide a benefit of passive targeting	Rheumatoid arthritis	^79^
4	Cancer cell membrane	PLGA NPs	HepG2 and RAW264 cell lines and BALB/C nude mice bearing HepG2 cells	Enhanced in vitro cellular endocytosis and antitumor effect toward HepG2 cells. Significantly enhanced in vivo antitumor effect and decreased systemic toxicity as compared to control (doxorubicin) owing to enhanced systemic circulation, enhanced doxorubicin accumulation at the tumor site and effective immune escape.	Cancer	^165^
5	Neural stem cell membrane	PLGA NPs	Male C57BL/6 mice bearing stroke through MCAO surgery	Enhanced delivery of NPs to the ischemic brain by membrane coating Greater chemotactic interaction of CXCR4 with SDF‐1 Increased the efficacy of glyburide, an anti‐edema agent	Stroke	^228^
6	Macrophage membrane	PLGA NPs	RAW264.7 cells pretreated with lipopolysaccharide and Tnfα recombinant protein and Mouse model of DSS‐induced ulcerative colitis	Significantly alleviated the symptoms of ulcerative colitis Achieved immunomodulatory and suppressive effects by reducing S100a9 and other cytokines in the colitis region	Ulcerative colitis	^273^
7	RBC membrane	PLGA NPs	Mice model infected with *Plasmodium yoelii* BY265	Prolonged circulation, enhanced antimalarial efficacy, targeted delivery to Plasmodium‐iRBCs, and satisfactory biocompatibility	Malaria	^274^
8	RBC membrane	PLGA NPs	Neuronal‐like cell line (HT22 cells), BBB model using Transwell plates and AD mice model	Relieved symptoms of AD by reducing p‐tau levels Suppressed neuronal‐like cells death both in vitro and in vivo Improved memory impairment in an AD mouse model	AD	^275^
9	RBC membrane	PLGA NPs	Chlorpyrifos as model organophosphate and rabbit model of organophosphate poisoning	Prevented AChE inhibition in dose dependent manner Complete recovery of AChE activity and no observable liver or kidney injury in rabbit model	Organophosphate poisoning	^276^
10	Hybrid cell‐membrane	PLGA NPs	Laser‐induced wet age‐related macular degeneration mouse model	Homotypic targeting and competitively binded to the VEGF	Choroidal neovascularization	^277^

Abbreviations: AD, Alzheimer disease; BBB, blood–brain barrier; DSS, dextran sulfate sodium; NPs, nanoparticles; PLGA, poly(lactic‐*co*‐glycolic acid); RBC, red blood cell; VEGF, vascular endothelial growth factor.

## 4 CURRENT CHALLENGES AND FUTURE DIRECTIONS

Although cell membrane‐coated PLGA NPs have shown promising results against different diseases, several challenges are still there, and further research and development are needed to address them and make the application of cell membrane‐coated PLGA NPs more widely accessible. Main challenges are related to cell membrane extraction, coating of PLGA NPs with cell membranes and the applications of cell membrane‐coated PLGA NPs in different disease conditions.

For cell membrane extraction from various cells, differential centrifugation and density gradient centrifugation methods are generally used.^278^ However, the centrifugation parameter and choice of cell fragmentation methods have not been clearly established yet. The stability of cell membrane without nutrient supply and cytoplasmic support is still an issue. The risk of bacterial contamination is another issue that may complicate platelet membrane, which are usually stored at 22–24°C for 5–7 days.^278^ Yield of coating of NPs with cell membranes is another challenge with cell membrane production, for example, for high yield of WBC, platelet and stem cell membranes are difficult to achieve. It has been estimated that to coat 1 mg of PLGA NPs, up to 3 × 10^9^ of platelets are needed. In this regard, a simplified method with high yield of cell membrane is urgently needed.^279^ Large‐scale manufacturing of cell membrane‐camouflaged NPs is a major limitation for wider use of coated NPs. Cell membrane coating technology is primarily dependent of membrane extrusion and sonication.^12^ Both these methods have pros and cons. For example, sonication is characterized by high production efficiency but low uniformity, while membrane extrusion has low production efficiency and high uniformity. Cell factories, fully automated cell culture systems can be good solutions for the issues related to cell membrane extraction. Future studies should focus on the combination of cell factory technology and existing cell membrane extraction strategies to develop assembly line for cell culture and cell membrane isolation. Microfluidics‐based approach could be a viable choice in future for high‐quality fabrication and facile scale‐up. Microfluidic electroporation^280^ and microfluidic sonication^135^ for membrane coating have proved better colloidal stability, complete membrane coating, and targeting efficiency.

In addition to cell membranes, there are also challenges related to PLGA drug carriers, despite their use already over 30 years. These are related to formulation‐related aspects pertaining to drug loading and release. One of the key issues related to fabrication of biosimilar PLGA‐based drug products is the complexity of the manufacturing process. Slight changes in the manufacturing process, involving quality control/assurance (QC) systems, can greatly influence the bioactivity, efficacy, safety, and stability of the product. Polymer degradation and drug release are greatly influenced by the physicochemical properties of PLGA such as molecular weight, particle size, porosity, drug polymer interaction, and glass transition temperature.^281^ The higher entrapment efficiency of water soluble drugs in hydrophobic PLGA matrix is the major limitation. Grafting PLGA with block polymers having amphiphilic sites will greatly improve the encapsulation efficiency of water soluble drugs.^281^ Drug loading, encapsulation, and release are significantly affected when one of emulsion‐based technique is switched to another one (O/W, W/O, and W/O/W).^282^ Because conventional methods suffer from limitations, such as small batch and large batch production can alter the formulation characteristics, new emulsification technologies, such as microfluidics‐based and membrane emulsification have been developed.^283^ The production and storage costs of NPs also need to be considered when patient's own cells are used. Therefore, continuous screening of these NPs for better biocompatibility, higher specificity, better method of preparation and wider applications should constitute a major future direction of research and development. The outcome from studies of cell membrane‐coated NPs is that we learn which proteins are essential for a given function and that we use this knowledge to enable the development of fully synthetic NPs that can conduct that function. There is a limited number of proteins needed for directed targeting and delivery of therapeutics, and functional elements of these proteins could be synthesized and attached to NPs to improve scale‐up, reduce costs and increase the self‐life.

Successful coating and orientation of cell membrane on NPs are critical issues that may arise during preparation of cell membrane‐camouflaged NPs. Effective coating of NPs using cell membranes is required to preserve cellular proteins and their functions. However, studies showed that up to 90% of the biomimetic NPs are only partially coated. The percentage of full coating is low with all the three methods, that is, 1.8 ± 0.1%, 6.2 ± 0.3%, and 6.5 ± 0.3% for sonication, extrusion, and combined sonication–extrusion, respectively, which shows the superior efficacy of extrusion process than sonication in the formation of a full cell membrane coating. The integrity of cell membrane coating affects the endocytic entry mechanism of coated NPs. NPs with a high degree of coating (>50%) display individualized cellular internalization, whereas those with low degree of coating (<50%) require aggregation before internalization.^284^ Partially coated NPs were found to enter cancer cells via a cooperation mechanism based on appropriate aggregation of NPs.^284^ Therefore, careful evaluation of the ratio of full cell membrane coating will enhance tumor targetability.

The asymmetric biological characteristics of cellular membranes, differences between the cytoplasmic and outer surfaces, means that the right‐side‐out orientation of cell membrane coating is also a vital for effective therapies. As seen in the case of RBC membrane coating on PLGA NPs, a strong negative charge exists on the extracellular side of RBC membrane than on the intracellular cytoplasmic side.^64^ Therefore, electrostatic interaction is favored between intracellular surface of RBC membrane and negatively charged PLGA NPs, which biased the right‐side‐out orientation. This mechanism will not benefit positively charged NPs.

For the development of versatile cell membrane NPs, certain modifications to the plasma membrane are inevitable and may induce undesirable adverse effects. For example, excessive use of immune cell membrane coating may interact with immune system, leading to release of pathological mediators and may induce inflammation.^12^ In case of platelet membrane coating, the pro‐inflammatory effect has been reported to promote the development of atherosclerosis.^285^ Biocompatibility is another concern of cell membrane‐coated NPs. Although short‐term biocompatibility has been demonstrated in different studies,^56^ still the cell membrane extraction should be undertaken with great care to avoid potential risks. Limitations related to manufacturing of cell membrane‐coated PLGA NPs can be overcome with the use of 3D printing, microfluidics and particle replication in non‐wetting templates (PRINT). Recently, PRINT technology has enabled precise manufacturing of engineered particles with uniform biodegradable material cores and independent control over their chemical composition, shape and size to impart desired therapeutic benefits to the product.^197^

Although many applications have been explored, there certain issues that need better understanding. For example, the mechanism of drug release from cell membrane‐coated NPs is still unclear.^178^ Moreover, in comparison to synthetic materials, the purity and quality of coated NPs are hard to control, and their safety is also uncertain especially for CCM coated NPs, which can induce a harmful immune response.^286^ The impact of physical and chemical properties of small molecule loaded PLGA NPs and membrane types on the cellular uptake and small molecule release should, therefore, be extensively investigated to enhance their clinical translation. QC of these NPs should also be a focus of future research.

In cancer therapy, limited clinical outcomes of the nanomedicines in humans are due to failure of EPR effect and other complex biological characteristics of the tumor. Tumors of small animals differ significantly from those of humans in many aspects, such as metabolic rate, growth of tumor and tumor to host volume ratio. Therefore, it is inappropriate to predict the retention effects of nanomedicines in small animal models to actual human samples as the human tumors do not grow as fast as in the case of small animals. Besides, elevated interstitial fluid pressure and complex ECM compositions of tumors affect the distribution and accumulation of nanomedicines in tumors. These barriers are likely to be addressed by using cell membrane coating technology. However, strategies must be considered while tailoring cell membrane vesicle for anticancer applications. First, the narrow size range of membrane vesicle preferably within 12–50 nm will decrease the hepatic uptake and enhance the penetration through leaky vasculature. Second, co‐loading/administration of vasodilators or constrictors with nanovesicles to modulate tumor blood flow will enhance the EPR effect. Third, the anchoring of ECM‐degrading enzymes, like hyaluronidase, collagenase, and so forth to membrane bilayer will augment the abnormal tumor ECM and can assist diffusion and spatial distribution of nanovesicles in the interstitial space.^287^

Regarding the use of cell membrane coating technology as anti‐inflammation therapy, obstacles still exist before clinical translation can be achieved. Ex vivo cell expansion may alter the cell phenotype. Accordingly, it would be difficult to control the quality and purity of the entire population. A standardized culture protocol should be established and validated without altering the desired phenotype to obtain functional cell membranes for coating. In order to target the inflammatory microenvironment, a simple, effective, and reproducible membrane surface engineering is; therefore, required.^288^

For CVD application of cell membrane‐coated PLGA NPs, a better understanding of phenotypic changes during disease progression and additional cell types associated with CVD can be used as membrane coating. Diagnostic markers or bioactive compound such as miRNA can be combined with cell membrane technology. The miRNA‐based therapies have the potential to reverse the atherosclerosis.^219^

For precise application of cell membrane coating technology in treatment of infectious, pathogen–host interaction is a key issue that needs further consideration. Strategies like genetic editing and bioconjugate chemistry can be employed for those pathogens with clear infection mechanisms to enhance the specific protein expression.^243^

In future, integrating newer technologies of cell membrane extraction, coating and standardization methods and methods to scale up will improve the outcome of using new cell membrane‐coated biomimetic PLGA NPs and help their translation to the clinic to enable addressing many of the current challenges related to inflammation, infection, cancer and other emerging health problems.

## 5 CONCLUSION

Cell membrane‐camouflaged NPs are core–shell structures that combine the intrinsic properties of natural cell membranes and synthetic NP cores and offer unique opportunities in biomedicine. Different cell surface proteins are expressed on the surface of cell membranes isolated from various sources, and these help to determine the affinity and accumulation of cells at sites of pathologies. Coating with cell membranes leads to prolonged NP circulation time, enables NP to evade immune cells and results in targeted drug delivery. Most used NP cores are composed of the biodegradable polymer, PLGA, largely because it is already approved by the FDA for many applications. PLGA nanoparticles can entrap both water soluble and insoluble drugs, protect the drugs from degradation, provide sustained release and can increase therapeutic efficacy by improving the pharmacokinetics and pharmacodynamics of the active pharmaceutical ingredients. Therefore, merging of natural cell membranes and synthetic PLGA NPs core is an exciting area of investigation that may lead to improved delivery of therapies for the treatment of pathological conditions such as cancer, inflammation, CVDs and microbial infections. Cells have much to teach us about targeted delivery and using their membranes on synthetic nanoparticles will lead to discovery of innovative approaches and new opportunities in medicine.

## AUTHOR CONTRIBUTIONS

**Nasrullah Jan:** Conceptualization (equal); visualization (supporting); writing – original draft (lead); writing – review and editing (supporting). **Asadullah Madni:** Conceptualization (supporting); writing – original draft (supporting); writing – review and editing (supporting). **Safiullah Khan:** Writing – original draft (supporting). **Hassan Shah:** Writing – original draft (supporting). **Faizan Akram:** Writing – original draft (supporting). **Arshad Khan:** Writing – original draft (supporting). **Derya Ertas:** Visualization (equal); writing – original draft (supporting); writing – review and editing (supporting). **Mohammad Fauzi Bostanudin:** Writing – review and editing (supporting). **Christopher H. Contag:** Writing – original draft (supporting); writing – review and editing (supporting). **Nureddin Ashammakhi:** Conceptualization (supporting); writing – original draft (supporting); writing – review and editing (equal). **Yavuz Nuri Ertas:** Conceptualization (lead); visualization (lead); writing – original draft (lead); writing – review and editing (lead).

## CONFLICT OF INTEREST

The authors declare no conflict of interest.

### PEER REVIEW

The peer review history for this article is available at https://publons.com/publon/10.1002/btm2.10441.

## Data Availability

The data that support the findings of this study are available from the corresponding author upon reasonable request.
